# Sex-Specific Effects of Chronic Creatine Supplementation on Hippocampal-Mediated Spatial Cognition in the 3xTg Mouse Model of Alzheimer’s Disease

**DOI:** 10.3390/nu12113589

**Published:** 2020-11-23

**Authors:** Wanda M. Snow, Chris Cadonic, Claudia Cortes-Perez, Aida Adlimoghaddam, Subir K. Roy Chowdhury, Ella Thomson, Adama Anozie, Michael J. Bernstein, Kathleen Gough, Paul Fernyhough, Miyoung Suh, Benedict C. Albensi

**Affiliations:** 1 Division of Neurodegenerative Disorders, St Boniface Hospital Albrechtsen Research Centre, Winnipeg, MB R2H2A6, Canada; ccadonic@sbrc.ca (C.C.); cperez@sbrc.ca (C.C.-P.); aadlimoghaddam@sbrc.ca (A.A.); skr_chowdhury@yahoo.ca (S.K.R.C.); ethomson@stanford.edu (E.T.); yanozie@gmail.com (A.A.); pfernyhough@sbrc.ca (P.F.); miyoung.suh@umanitoba.ca (M.S.); 2 Centre for the Advancement of Teaching and Learning, University of Manitoba, Winnipeg, MB R3T 2N2, Canada; 3 Research Institute in Oncology, CancerCare Manitoba, University of Manitoba, Winnipeg, MB R3T 2N2, Canada; 4 Department of Psychological and Social Sciences, Pennsylvania State University Abington, Abington, PA 19001, USA; mjb70@psu.edu; 5 Department of Chemistry, University of Manitoba, Winnipeg, MB R3T 2N2, Canada; kathleen.gough@umanitoba.ca; 6 Department of Pharmacology & Therapeutics, University of Manitoba, Winnipeg, MB R3T 2N2, Canada; 7 Department of Human Nutritional Sciences, University of Manitoba, Winnipeg, MB R3T 2N2, Canada

**Keywords:** creatine, Alzheimer’s disease, hippocampus, 3xTg model, Morris water maze, nuclear factor kappa b

## Abstract

The creatine (Cr) energy system has been implicated in Alzheimer’s disease (AD), including reductions in brain phosphoCr and Cr kinase, yet no studies have examined the neurobehavioral effects of Cr supplementation in AD, including the 3xTg mouse model. This studied investigated the effects of Cr supplementation on spatial cognition, plasticity- and disease-related protein levels, and mitochondrial function in the 3xTg hippocampus. Here, 3xTg mice were fed a control or Cr-supplemented (3% Cr (*w/w*)) diet for 8–9 weeks and tested in the Morris water maze. Mitochondrial oxygen consumption (Seahorse) and protein levels (Western blots) were measured in the hippocampus in subsets of mice. Overall, 3xTg females exhibited impaired memory as compared to males. In females, Cr supplementation decreased escape latency and was associated with increased spatial search strategy use. In males, Cr supplementation decreased the use of spatial search strategies. Pilot data indicated mitochondrial enhancements with Cr supplementation in both sexes. In females, Cr supplementation increased CREB phosphorylation and levels of IκB (NF-κB suppressor), CaMKII, PSD-95, and high-molecular-weight amyloid β (Aβ) species, whereas Aβ trimers were reduced. These data suggest a beneficial preventative effect of Cr supplementation in females and warrant caution against Cr supplementation in males in the AD-like brain.

## 1. Introduction

**Figure 1:**
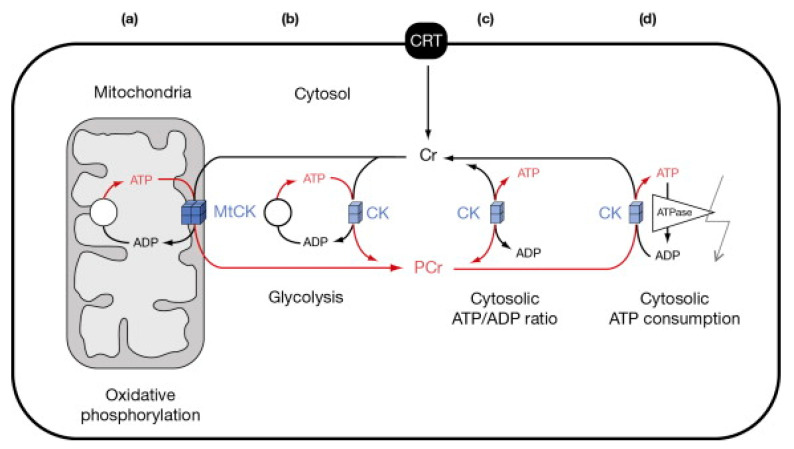
“The creatine kinase (CK)/phosphocreatine (PCr) system. Isoenzymes of CK (blue) are found in different compartments such as mitochondria (octameric MtCK, (**a**)) and cytosol (dimeric CK, (**b**)–(**d**)) in soluble form (**c**) or associated to a different degree to ATP-delivering ((**a**) and (**b**)) or -consuming processes (**d**). A large cytosolic PCr pool up to 30 mM is built up by CK using ATP from oxidative phosphorylation like in heart (**a**) or glycolysis like in fast-twitch glycolytic muscle (**b**). PCr is then used to buffer global (**c**) and local (**d**) ATP/ADP ratios. In cells that are polarized and/or have very high or localized ATP consumption, these CK isoenzymes, together with easily diffusible PCr, also maintain an energy shuttle between ATP-providing or -consuming processes ((**a**) and (**d**)). Metabolite channeling occurs where CK is associated with ATP-providing or -consuming transporters, pumps, or enzymes ((**a**), (**b**), and (**d**)). Creatine (Cr) is synthesized in only few cell types (e.g., liver and kidney) and has to be taken up from the blood stream by a specific Cr transporter (CRT) that is highly expressed by Cr-containing target cells.” Figure and legend reprinted from [4] Encyclopedia of Biological Chemistry, Vol 2, Schlattner, U., Tokarska-Schlattner, M., and Wallimann, T. Metabolite channeling: creatine kinase micro compartments, 80–85, Copyright (2013), with permission from Elsevier Publishing Company. http://www.elsevier.com.

Creatine (Cr) has been well accepted as an effector in muscle, however several lines of evidence suggest a role for Cr in cognition (as reviewed in [1,2]). An endogenous amino acid, Cr is synthesized from glycine, arginine, and S-adenosylmethionine in several organs, including the brain [3]. The enzyme Cr kinase (CrK) catalyzes the conversion of Cr and ATP to phosphoCr (pCr) and adenosine diphosphate (ADP) in a reversible fashion (see Figure 1 [4]). In cells, Cr is transported across the plasma membrane by the Na^+^- and Cl^-^-dependent Cr transporter (CRT) [5]. PCr is a high-energy reserve molecule that is important for energy homeostasis in tissues with fluctuating energy demands, such as the brain.

In addition to being an important regulator of cellular energy status, Cr has been purported to possess neuroprotective properties. In one study, treatment of rat hippocampal cultures with Cr protected against amyloid β (Aβ) toxicity for up to 24 h [6]. Reduction in ATP occurs early in the process of Aβ-induced toxicity [7]. Cr treatment, however, elevates ATP levels in hippocampal neuronal cultures [6]. ATP levels are important determinants of whether a cell will undergo apoptotic or necrotic changes in response to excess Ca++ [8]. In neurons, pCr is also elevated with Cr treatment alone or in the presence of Aβ [6]. Thus, exogenous Cr is capable of regulating several parameters of neuronal bioenergetics in both physiological and pathological conditions in vitro. In Alzheimer’s disease (AD), the neurodegenerative disorder is characterized by severe cognitive dysfunction and memory impairments, neuron loss, and deposition of plaques comprised of Aβ peptides. These changes are robust and occur years before cognitive deficits are clinically detectable [9]. The reported ability of Cr to regulate Aβ-induced toxicity, therefore, hints at the possible therapeutic or preventative potential of Cr in AD.

In addition to alterations to Aβ processing, perturbations to the transcription factor nuclear factor kappa b (NF-κB) have been consistently reported in AD [10], although inconsistencies exist regarding the direction and magnitude of these alterations. For instance, although several studies confirm elevations in NF-κB subunits [11,12,13,14], others report depressed levels of NF-κB in the nuclei in activated form [15], suggesting abnormalities with nuclear translocation or activation of NF-κB in the disease. Although classically implicated in inflammation and cancer biology, NF-κB is also implicated in normal learning, memory, and synaptic function [16]. The effects of NF-κB in the brain are cell-specific; in neurons, NF-κB activation induces expression of genes promoting plasticity and cell survival, whereas in glia, activation exerts a pro-inflammatory effect (as reviewed in [17]). Several effectors and targets of NF-κB in neurons are also associated with AD pathology, including the amyloid precursor protein (APP) and Aβ (as reviewed in [10]). In vitro, the neuroprotective effects of Cr appear to be mediated by NF-κB-dependent pathways, as treatment of cultured neurons with Cr reduced levels of IκB, the NF-κB tethering protein, consistent with enhanced activation of NF-κB with Cr [18]. Consistent with such data, our previous work with Cr supplementation in C57BL/6 mouse model [19] demonstrated a similar reduction in hippocampal IκB protein levels, consistent with increased activation of NF-κB, as well as elevations in calcium-calmodulin-dependent protein kinase II (CaMKII), a protein shown to be required for long-term memory formation [20] and for which isoforms serve as both activators [21] and downstream targets of NF-κB [22]. Further, hippocampal levels of postsynaptic density protein 95 (PSD-95), a synaptic protein implicated in learning and memory that is also under the transcription regulation of neuronal NF-κB [23], were increased in Cr-supplemented mice. Importantly, these molecular alterations were accompanied by enhanced hippocampal-associated learning, memory, and mitochondrial function [19]. Moreover, a subunit of complex I of the electron transport chain, which is intricately involved in mitochondrial function, was elevated with oral Cr [19]. The effects of oral Cr, however, on learning, memory, NF-κB-related protein levels, and mitochondrial function have not been examined in an AD model.

To date, there are no published clinical studies examining the effects of oral Cr in AD patients, despite evidence of dysfunction in the brain Cr system in the disease. For example, both upregulation [24] and downregulation [25] of brain pCr have been reported in early AD. Further, the brain isoform of CrK, which catalyzes the conversion of Cr to PCr, is severely reduced in the brains of those with AD relative to healthy age-matched control subjects [26]. In the brains of those carrying the ε4 allele of the ApoE gene, one of the few identified genetic risk factors for late-onset AD, brain Cr levels were significantly lowered and were correlated with the degree of cognitive impairment [27].

Several animal models of AD have been developed to help elucidate the pathological underpinnings of AD-related cognitive dysfunction and to test putative treatments and prophylactic measures. At present, only a limited number of studies investigating the possible benefits of Cr supplementation, the most clinically relevant route of administration, have been performed in animal models of AD. In one study, performance in the Morris water maze (MWM), a test of hippocampal-mediated spatial cognition, was not improved with oral Cr (2%) in rats exposed to a single Aβ intrahippocampal injection, nor was the degree of hippocampal Aβ-induced apoptosis [28]. In Tg2576 mice, however, the same dose protected against AD-related neuropathology, including reduced brain weight, increased Aβ plaque load, and decreased ATP levels [29]. The bioenergetic and behavioral ramifications of Cr supplementation have not been examined in a model of the disease with another key pathological feature, neurofibrillary tangles (NFT). Of note is the finding that injection of oligomers of tau, the protein comprising NFT, reduced levels of complex I protein subunits in mouse brain tissue [30]. In mice overexpressing tau, reductions in complex I activity and protein levels are noted in conjunction with deficits in mitochondrial respiration [31]. Mitochondrial deficits have been consistently reported in AD [32], including impairments in the electron transport chain (ETC) complexes [33], including complex I [34], which transfers electrons from nicotinamide adenine dinucleotide + hydrogen (NADH) to coenzyme Q within the ETC. Thus, Cr supplementation may offer therapeutic benefit in AD due its ability to increase complex I levels and enhance oxidative respiration in brain mitochondria [19].

The present study sought to determine the effects of chronic oral Cr consumption (3%, *w/w*) on spatial memory in a mouse model of AD that possesses two of the cardinal neuropathological features of AD (Aβ plaque deposition and NFT), the 3xTg strain [35]. As sex differences have been noted both in this strain [36], in the therapeutic effects of Cr supplementation [37,38], and in AD [39], experiments and analyses were conducted, taking sex into account. This study examined the effects of oral Cr specifically in the hippocampus, an area particularly vulnerable to AD-related pathology and a key structure implicated in memory. Specifically, the effects of Cr supplementation on the hippocampal-dependent MWM, hippocampal Cr levels, and protein levels of key proteins implicated in AD, including APP, tau (total and hyperphosphorylated tau (ptau)), and NF-κB, were examined. Mitochondrial function was also assessed in mitochondria isolated from the hippocampus in mice supplemented with Cr compared to control-fed mice. These experiments revealed sex-dependent cognitive enhancements with Cr supplementation in the 3xTg model, with females demonstrating improved spatial cognition relative to males, despite pilot results indicating mitochondrial enhancements in both sexes. Further probing in females indicated upregulation of several plasticity-related proteins, as well as altered levels of several Aβ species in the hippocampus, indicating that Cr supplementation can positively impact both behavioral and neuropathological correlates of AD in females in this widely used mouse model.

## 2. Materials and Methods

### 2.1. Animals, Dietary Supplementation, and Experimental Design

Experiments were conducted in male and female 3xTg mice, as described in detail elsewhere [40]. Mice were randomly assigned to either a control or Cr-supplemented diet at 7 months of age and kept on the diet for 8 weeks ± 7 days based on the experimental endpoint. Cr monohydrate (3%, *w/w*, Alfa Aesar, Ward Hill, MA, USA) was mixed with a semi-synthetic nutritionally complete diet, as described elsewhere [19]. Food and water were provided ad libitum. Weight was measured weekly, whereas food intake was measured twice weekly after subtracting the amount of food remaining from the food provided. Testing in the MWM began at week 8, followed by mitochondrial experiments at week 9. During week 6 of supplementation, one male Cr-fed mouse developed a prolapsed penis that was not responsive to treatment and was euthanized. All data from this mouse were excluded from any analysis. The resultant sample sizes from *N* = 24 mice for behavioral tests were: control: *n* = 6 males and *n* = 6 females; Cr-supplemented diet: *n* = 5 males and *n* = 8 females.

Tissue for Western blot experiments was collected after MWM testing. Mice were euthanized by decapitation under anesthetic intraperitoneal injection of ketamine at 62.5 mg/kg and xylazine at 12.5 mg/kg after MWM testing. After brain extraction, the hippocampi were dissected, snap-frozen in liquid nitrogen, and stored at −80 °C. For functional mitochondrial assays, mice were euthanized under anesthetic (isoflurane inhalation) and the hippocampi were removed for immediate mitochondrial isolation.

**Figure 2:**
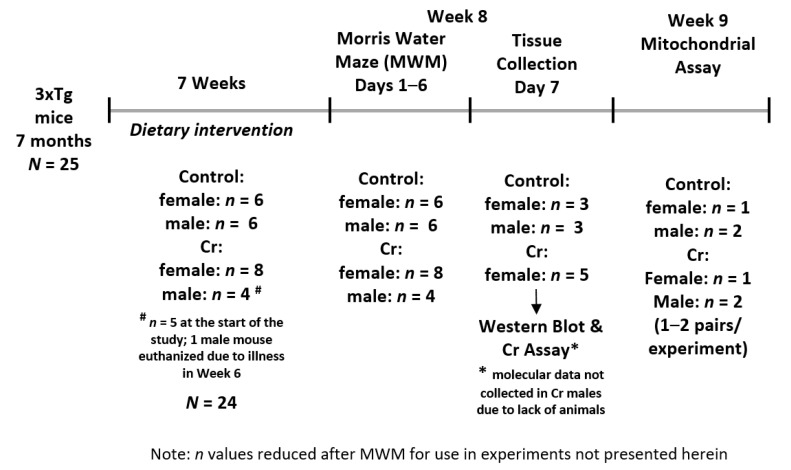
Experimental timeline of the study.

All experiments reported herein were conducted at St. Boniface Hospital Albrechtsen Research Center. Mice were housed in standard cages individually at the animal holding facility and were kept on a 12-h light/12-h dark schedule at 22 °C and 40% humidity. All experiments were approved by the University of Manitoba Animal Care and Use committee (Ethics approval #09-026/3), which conforms to the Canadian Council for Animal Care’s Guide to the Care and Use of Experimental Animals. Figure 2 depicts the experimental timeline of the study.

### 2.2. Morris Water Maze (MWM)

Hippocampal-associated spatial learning and memory were evaluated with the MWM, as previously described [40]. The apparatus consisted of a circular pool (100-cm diameter) filled with tap water (24–25 °C), which was made opaque with non-toxic white paint. A platform (10 cm) was submerged approximately ~5 mm below the water line. Visual cues were placed on the interior wall of the maze equidistant from the water level and above the water level, whereas non-maze cues were blocked from view using a screen. Mice were trained to locate the platform for 5 days with 3 trials per day, each 90 s long. On the first day, in cases where the platform was not located after the 90-s trial, mice were placed on the platform for 10 s before removal. Escape latency and swim speed values were calculated from acquisition data. As the reliance on different search strategies changes over the course of training in the MWM and can indicate evidence of forming a spatial map [41], the frequency of use of search strategies was also analyzed. Search strategies were determined from path traces during acquisition trials and categorized as one of nine strategies under three broad categories, as per [42]: (1) repetitive looping: (a) peripheral looping: swimming along the outer edge of the pool wall; (b) chaining: swimming around the pool at an approximately fixed distance from the pool wall within the pool’s interior; (c) circling: swimming in a tight circular formation; (2) non-spatial systemic: (a) scanning: swimming in the interior pool area with no apparent pattern; (b) random: swimming the entire pool with no apparent pattern; (c) focal incorrect: swimming within a specific quadrant outside of the target quadrant; (3) spatial: (a) spatial direct: swimming directly to the platform; (b) spatial indirect: swimming to the platform with no more than one loop; (c) focal correct: direct swimming to and searching confined to the correct quadrant. When more than one search strategy was used, the trial was classified according to the dominant strategy used.

Parameters to elucidate memory retention were calculated from a 90-s trial on the 6th day with the platform removed, and included time spent and number of entries into the target quadrant, as well as the number of passes over the platform area.

On each day of training, the order in which mice were exposed to the MWM was randomly assigned using a random number generator. The initial starting quadrant for the first trial of the first day was also randomly determined, after which time mice entered the pool on the next clockwise quadrant on each subsequent trial. Starting positions on subsequent days were moved to the next clockwise quadrant, such that overall each mouse entered the pool from each of the three non-target quadrants once per day.

### 2.3. Protein Extraction

After storage at −80 °C, brain tissue was placed in cold radioimmunoprecipitation assay (RIPA) buffer (150 mM sodium chloride, 1.0% Triton X-100, 0.5% sodium deoxycholate, 0.1% sodium dodecyl sulphate (SDS), and 50 mM Tris, pH 8.0) with 1% protease inhibitor cocktail (Amresco, Solon, OH, USA) and 1% phosphatase inhibitor cocktail (Sigma-Aldrich, St. Louis, MO, USA), then agitated for 1 h at 4 °C. After lysing, tissue was centrifuged at 10,000× *g* for 10 min at 4 °C. Supernatants were collected and protein concentrations were measured with the DC Protein Assay (Bio-Rad, Hercules, CA, USA). Samples were then diluted in RIPA buffer to equivalent protein concentrations. Given the reduced sample of male 3xTg mice supplemented with Cr after MWM testing, all data using hippocampal homogenates were conducted in only three of the four groups (male and female control fed mice; Cr-supplemented female 3xTg mice). This still allowed for comparisons between sex in the 3xTg strain and between diet in 3xTg females, in which dietary supplementation improved performance in the MWM.

### 2.4. Western Blots

Hippocampal homogenates were diluted further in 4x Laemmli buffer (16% SDS, 40% glycerol, 20% β-mercaptoethanol, 0.01% bromophenol blue, and 0.25M Tris, pH 6.8) before denaturing at 50 °C for 8 min. Equivalent protein amounts (10–20 µgs) per sample were loaded onto polyacrylamide gels (either Invitrogen, Novex™ 4–20% Tris-Glycine Mini Gels, WedgeWell™ format, Carlsbad, CA, USA or Criterion™ TGX Stain-Free™ gels, Bio-Rad), followed by SDS-PAGE (200 V for 45 min). For Stain-Free™ gels, gels were activated using a Bio-Rad UV transilluminator (ChemiDoc™ MP Imaging System) to enable visualization of total protein bands. Proteins were then transferred to nitrocellulose membranes (Bio-Rad, Hercules, CA, USA) using the Trans-Blot^®^ Turbo™ Transfer System (Bio-Rad, Hercules, CA, USA). Total protein was detected using either the ChemiDoc™ MP alone (Stain-Free™) or with reversible Ponceau S staining. Membranes were blocked for 1 h at room temperature in 1X Tris-buffered saline with 0.1% Tween-20 (TBS-T) with 5% skim milk to reduce non-specific binding. In membranes exposed to antibodies against cAMP response element-binding protein (CREB) in its phosphorylated form (pCREB), 5% bovine serum albumin was used as an alternative to milk. After blocking, membranes were incubated in primary antibodies (see Table 1) in a vehicle of TBS-T + 5% skim milk at 4 °C overnight, followed by washing in TBS-T. Membranes were then incubated in either peroxidase-conjugated AffiniPure goat antirabbit or goat antimouse IgG (H + L) antibody (1:5000–1:7500 dilution used, depending on the primary antibody; Jackson ImmunoResearch Laboratories, West Grove, PA, USA) for 1.5 h at 4 °C. Bands were detected using enhanced chemiluminescence (ECL) with Bio-Rad Clarity™ Western ECL Blotting Substrate (Bio-Rad, Hercules, CA, USA) and imaged with the ChemiDoc™ MP (Bio-Rad, Hercules, CA, USA). Densitometry was semi-quantified using ImageLab™ software, with values normalized to total protein. Densitometry values from control-fed female mice served as reference controls. Membranes exposed to pCREB antibody were stripped and re-probed with the rabbit monoclonal anti-CREB primary antibody (Table 1). Membranes exposed to AT8 antibody against ptau were stripped and re-probed with a primary antibody against total tau (Table 1).

### 2.5. Creatine Assay

The levels of Cr in hippocampal homogenates were measured using the Creatine Colorimetric Assay Kit (BioVision Inc., Milpitas, CA, USA), as per the manufacturer’s instructions. Cr was measured from 10 μL of homogenate diluted to a concentration of 4 mg/mL of protein.

### 2.6. Mitochondrial Oxygen Consumption Rates from Freshly Isolated Hippocampal Mitochondria

After MWM testing, the hippocampi were extracted and mitochondria were freshly isolated from a subset of animals with a differential centrifugation method [43]. Briefly, hippocampi were placed in mitochondrial isolation buffer consisting of 70 mM sucrose, 210 mM mannitol, 5 mM HEPES, 1 mM EGTA, and 0.5% (*w/v*) fatty-acid-free bovine serum albumin (BSA) at pH 7.2 and homogenized manually. After centrifugation of the homogenate (800× *g* for 10 min, 4 °C), the pellet was removed and the supernatant was spun (twice at 800× *g* for 10 min at 4 °C, once at 8000× *g* for 15 min at 4 °C). Mitochondrial isolation buffer was added to the remaining pellet and spun (8000× *g* for 15 min at 4 °C), followed by resuspension in phosphate-buffered saline. Protein concentrations were measured using the DC Protein Assay (Bio-Rad, Hercules, CA, USA), as per manufacturer instructions.

Oxygen consumption rates (OCR) were measured in real time from isolated hippocampal mitochondria using the Seahorse XF Analyzer (Agilent Technologies, Santa Clara, CA, USA). Mitochondrial protein (10 μg/well) was diluted in mitochondrial assay solution (70 mM sucrose, 220 mM mannitol, 10 mM, KH2PO4, 5 mM MgCl2, 2 mM HEPES, 1 mM EGTA, and 0.2% BSA) with the addition of pyruvate (10 mM) and malate (2 mM), followed by plating (final volume of 450 uL) onto a Seahorse plate. After measuring basal respiration rates, coupled respiration was measured following the addition of ADP (4 mM) as an ATP synthase substrate and calculated after subtraction of basal OCR. Next, oligomycin (2 μM) was added to inhibit ATP synthase activity, ceasing coupled respiration, followed by addition of the protonophore–uncoupler carbonyl cyanide *p*-trifluoromethoxy-phenylhydrazone (FCCP; 2–4 μM) to determine maximal respiration capacity. Lastly, rotenone (Complex I inhibitor) and antimycin (Complex III inhibitor) were added (2 μM each) to prevent mitochondrial respiration. OCR in the presence of ADP divided by basal OCR was considered a measure of coupling efficiency. The coupled respiratory control ratio (RCR) was determined by dividing OCR with ADP by OCR with oligomycin. The maximal uncoupled RCR was calculated by dividing OCR with FCCP by OCR with oligomycin. Non-mitochondrial OCR (after rotenone and antimycin) values were subtracted from calculations of mitochondrial parameters. In males, the average value for each measurement from *n* = 2 mice was used.

### 2.7. Statistical Analysis

Food intake was calculated by averaging the mg of food consumed per week, which was then divided by the animal’s body weight (g) to give a measure of mg/day/g of body weight in order to account for differences in food intake as a function of differences in body weight. Food intake was analyzed with a three-way ANOVA (2 (Sex) × 2 (Diet) × 8 (Week)), with Week as a repeated measure. MWM acquisition data were also analyzed using three-way ANOVAs (2 (Sex) × 2 (Diet) × 5 (Day)), with Day as a repeated measure. Pairwise comparisons were conducted using least significant difference tests. MWM retention phase data and weight gain were analyzed by two-way ANOVA (Sex × Diet) or Mann-Whitney U (MWU) tests when normality assumptions were not met. In such cases, relevant pairwise comparisons were conducted between the four groups and the Holm’s sequential Bonferroni correction was applied to maintain the family-wise type 1 error rate for such comparisons at *p* < 0.05. Spatial strategy usage data were analyzed by χ^2^ tests and Fisher’s exact tests. Mitochondrial data were analyzed by Student’s *t*-test or MWU when data were not normally distributed. Western blot data were analyzed using one-way ANOVA or Kruskal-Wallis (KW) tests when data were not normally distributed. In cases of unequal variance, Brown-Forsythe (BF) tests were used, followed by Games-Howell post hoc tests for pairwise comparisons. The strength of associations between hippocampal Cr levels and MWM memory parameters and between Cr levels and Western blot densitometry values were measured using Pearson correlation coefficients. All analyses were two-tailed and were carried out using SPSS V26 (IBM Corp, Armonk, NY, USA).

## 3. Results

### 3.1. Food Intake and Weight Gain

**Figure 3:**
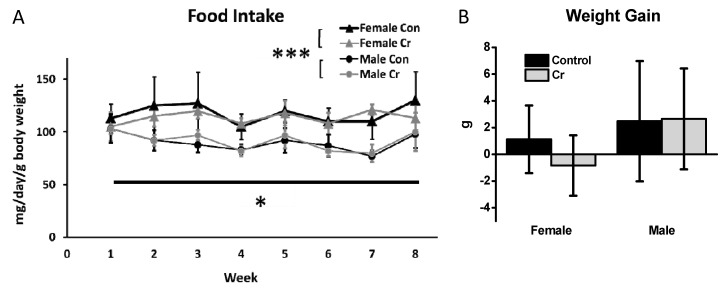
Food intake and weight gain as a function of diet in 3xTg mice. (**A**) Plot depicting food intake in adult (7-month-old) 3xTg control mice and 3xTg mice supplemented with Cr. Food intake (mg) was measured twice/week over the 8-week dietary intervention, averaged across the week, and expressed as mg/day. The mg/day value was divided by the animal’s body weight (g) to account for variations in food intake based on body weight. (**B**) Weight gain (ratio of g gained relative to initial body weight) over the 8-week study. Data were analyzed by two-way ANOVA. Mean ± SD depicted. Female control: *n* = 6; female Cr: *n* = 8; male control: *n* = 6; male Cr: *n* = 4; * *p* < 0.05; *** *p* < 0.001.

Analyses were conducted using mixed-model ANOVA, with Sex (2) × Diet (2) as between-subjects factors and Week (8) as a within-subjects factor. The 2 Sex × 2 Diet × 8 Week mixed-model ANOVA of food intake indicated significant main effects of Week (F(7140) = 3.1, *p* < 0.01) and of Sex (F(1, 20) = 40.67, *p* < 0.001). None of the interactions were significant, nor was there a significant main effect of diet (F(1, 20) = 0.09, *p* = 0.8). Over the course of the study, female 3xTg mice tended to eat more than did male 3xTg mice (91 ± 9.5 mg vs. 115 ± 7.5 mg, respectively), regardless of diet (Figure 3A).

Two-way ANOVA results for weight gain (calculated as a ratio of grams gained relative to starting body weight to rule out differences due to higher initial body mass) were not significantly different as a function of Sex (F(1, 20) = 3.14, *p* = 0.09) or Diet (F(1, 20) = 0.43, *p* = 0.5), nor was there a significant interaction effect (F(1, 20) = 0.6, *p* = 0.4). The approximate weight gain relative to starting body weight across all groups was 1.06 ± 3.38 g, however the results were highly variable amongst animals (Figure 3B).

### 3.2. MWM Parameters: Acquisition Phase

#### 3.2.1. Escape Latency

**Figure 4:**
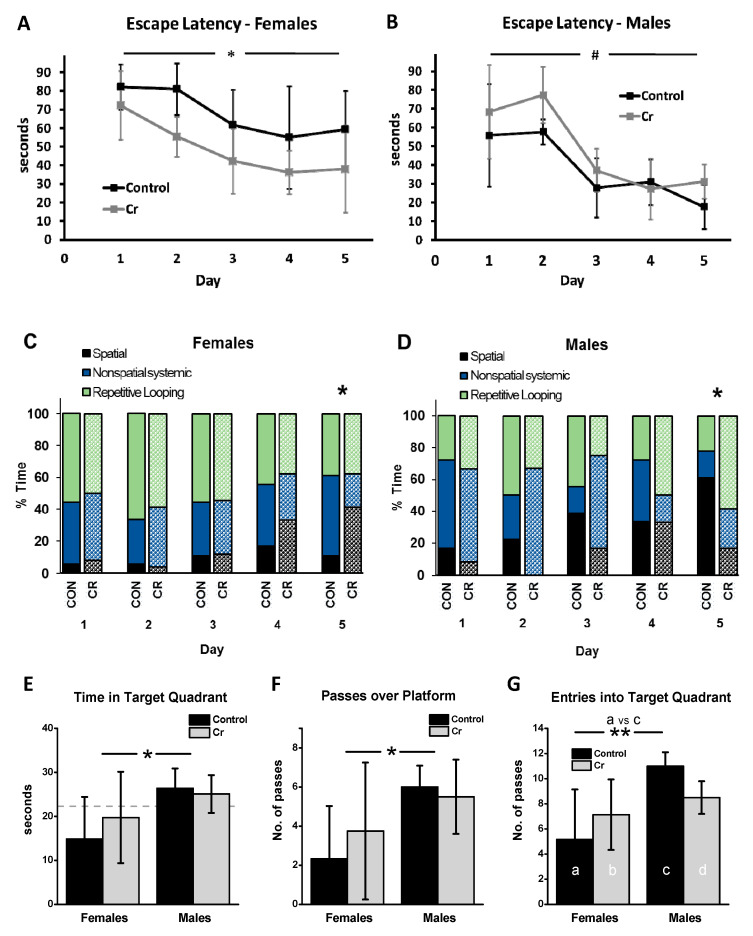
MWM (Morris water maze) as a function of diet in 3xTg mice. (**A**–**D**) Acquisition phase parameters as a function of diet in 3xTg mice. (**A**) In female mice, Cr supplementation significantly decreased escape latency (time to locate the platform; mean ± SD) overall across training days, indicating enhanced spatial earning; two-way ANOVA. (**B**) In males, however, Cr supplementation did not enhance performance in the MWM during training, as measured by escape latency, with a trend towards significantly greater escape latencies in supplemented males. (**C**,**D**) The frequency of use (% of total) of various search strategies (operationally defined as per [42]) was calculated across training days for control (solid bars) and Cr-supplemented (hatched bars) 3xTg mice and analyzed using Chi-square tests. (**C**) In females, reliance on spatial strategies increased with training in Cr-supplemented females, with the frequency of use peaking by the end of training, an effect that was not seen in control-fed 3xTg females. (**D**) In males, however, a reverse pattern was noted, where Cr supplementation resulted in a decreased reliance on spatial strategies relative to those on the control diet. (**E**–**G**). Memory retention in the MWM. On the last (6th) day of training, the platform was removed and mice were allowed 90 s to swim. Memory retention (mean ± SD) was evaluated in control-fed and Cr-fed mice by measuring (**E**) time in the target quadrant, (**F**) passes over the platform area, and (**G**) entries into the target quadrant. Female mice, regardless of diet, spent less time in the target quadrant (at levels below chance; indicated by dotted line in E) and made fewer passes over the platform (**F**) area than male 3xTg mice, confirming sex differences in spatial memory in this model. (**G**) Cr supplementation did not alter the number of entries in the target quadrant in either males or females. Sex differences in the number of entries in the target quadrant were only evident in control-fed 3xTg mice. Two-way ANOVA or MW: female control: *n* = 6; female Cr: *n* = 8; male control: *n* = 6; male Cr: *n* = 4; * *p* < 0.05; ** *p* < 0.01; ^#^ *p* < 0.1.

A 2 Sex × 2 Diet × 5 Day mixed model ANOVA with Day as the within-subjects factor revealed a significant main effect of Day (F(4, 80) = 24.40, *p* < 0.001), with no significant interaction effects for Day. The escape latency significantly decreased over the course of training and was significantly lower at day 1 (69.97 s ± 21.75) vs. days 3 (42.68 s ± 20.01; *p* < 0.001), 4 (38.2 ± 19.5 s, *p* < 0.001), and 5 (37.14 s ± 23.15; *p* < 0.001). The main effect of Sex was significant (F(1, 20) = 13.36, *p* = 0.002); irrespective of diet, females (58.36 s ± 15.56) performed worse overall in the MWM relative to males (43.16 s ± 10.08) in the 3xTg strain. Although the main effect of Diet was not significant in the three-way ANOVA (F(1, 20) = 1.09, *p* = 0.3), the Diet × Sex interaction was significant (F(1, 20) = 12.35, *p* = 0.002). Post hoc two-way ANOVA of Day × Diet for each sex separately revealed that Cr significantly improved the performance of 3xTg females, as measured by a 28% decrease in escape latency (67.84 ± 11.16 vs. 48.88 ± 11.16 s; F(1, 12) = 9.9, *p* = 0.008) (Figure 4A). In contrast, Cr-fed male 3xTg mice took 27% longer to find the platform over the course of training relative to control-fed males (48.29 ± 7.56 s vs. 38.03 ± 7.56 s, respectively), although the effect was only marginally significant (F(1, 8) = 4.42, *p* = 0.07), with no interaction between Diet and Day (F(1, 8) = 0.50, *p* = 0.5) (Figure 4B).

#### 3.2.2. Search Strategy

In addition to escape latency, the frequency of use of various search strategies was analyzed during the acquisition phase. On the first day of training, no significant differences in the frequency of spatial versus all other strategies combined (non-spatial and repetitive looping) were found as a function of diet in either male (*p* = 0.6) or female (*p* = 1.0; Fisher’s exact tests) 3xTg mice. By the end of training, the frequency of use of spatial strategies was significantly higher (nearly 400%) in Cr-fed females than in controls (*p* = 0.04). In males, the frequency of use of spatial strategies was also significantly different between dietary groups but was much higher in control versus Cr-fed mice (*p* = 0.03). By day 5, spatial strategies were the predominant search strategy category employed by control-fed males. In contrast, Cr-supplemented males relied most heavily on repetitive looping strategies (Figure 4C,D).

#### 3.2.3. Swim Speed

To evaluate the possible involvement of enhanced motor abilities in the MWM as a confounding factor in the interpretation of latency parameters, swim speed was analyzed. Three-way ANOVA indicated a main effect of Day (F(4, 80) = 4.4, *p* = 0.003). Post hoc comparisons indicated that this was driven by a peak in swim speed on day 3 relative to all other days (vs. day 1: *p* < 0.05; vs. day 2: *p* < 0.05; vs. day 4: *p* < 0.01; vs. day 5: *p* < 0.05). The main effects of Diet (*p* = 0.5) and Sex (*p* = 0.4) were not significant, nor were any of the interaction effects (*p* = 0.1–0.97) (not shown).

### 3.3. MWM Parameters: Retention Phase

Various parameters were measured during the 90-s probe trial on day 6 of the MWM, in which the platform was removed. Two-way ANOVA of the time spent in the target quadrant indicated a significant main effect of sex (F(1, 20) = 5.86, *p* = 0.025), with no significant main effect of Diet (F(1, 20) = 0.27, *p* = 0.6) or interaction effect (F(1, 20) = 0.79, *p* = 0.4). Irrespective of diet, female 3xTg mice spent significantly less time in the target quadrant than male 3xTg mice, with only male mice performing above chance levels (Figure 4E).

Examination of the number of passes over the platform area found no significant effect of Diet (F(1, 20) = 0.17, *p* = 0.7), although the effect of Sex was significant (F(1, 20) = 6.01, *p* = 0.024), with no interaction effect (F(1, 20) = 0.8, *p* = 0.4). Overall, the number of passes over the platform area was significantly reduced in female relative to male 3xTg mice (Figure 4F).

As the number of entries into the target quadrant did not meet assumptions for parametric tests, MWU tests were conducted between relevant pairs of the four groups to allow for effects of sex and diet to be examined. Among control-fed mice, females made less than half the number of entries into the target quadrant relative to males (MWU: *p* < 0.009), indicative of impaired spatial memory of the platform location in females relative to males. In male mice, although Cr supplementation resulted in 22.7% fewer entries in the target quadrant relative to control-fed males in absolute terms, this was not significant after applying the Holm’s Bonferroni correction (MWU: *p* = 0.019; critical *p* < 0.0167). Diet had no significant effect on the number of entries into the target quadrant in female 3xTg mice (MWU: *p* = 0.3). Unlike the sex differences seen in control-fed 3xTg mice on this parameter, no significant differences were found between Cr-fed male and female 3xTg mice (MWU: *p* = 0.5) (Figure 4G).

### 3.4. Hippocampal Mitochondrial Analysis

**Figure 5:**
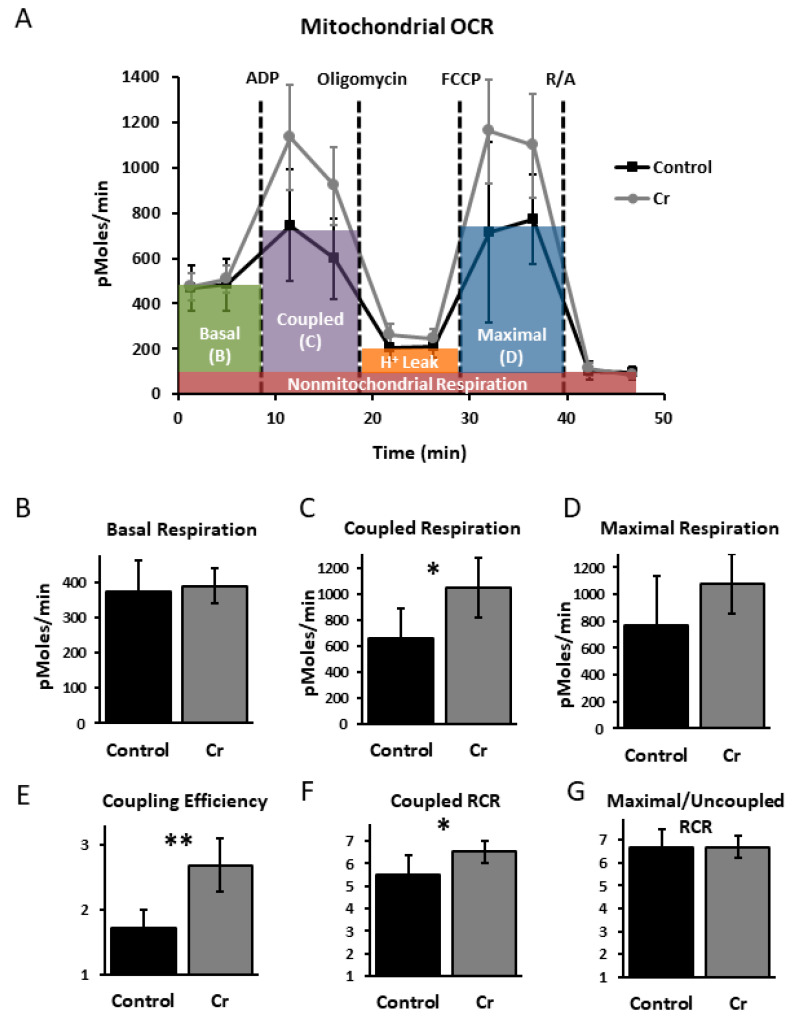
Oxygen consumption rates (OCR) in mitochondria isolated from the hippocampus in control and Cr-supplemented 3xTg mice. (**A**) Representative kinetics graph indicating real-time OCR at baseline and after addition of substrates adenosine diphosphate (ADP), oligomycin, carbonyl cyanide *p*-trifluoromethoxy-phenylhydrazone (FCCP), and rotenone-antimycin (R/A) in 10 μg of mitochondrial protein/well. (**B**) Basal respiration, (**C**) coupled respiration, and (**D**) maximal respiration (mean ± SD) were calculated after subtracting non-mitochondrial respiration (after R/A) and compared between control and Cr-supplemented mice. (**E**) Coupling efficiency was calculated by dividing coupled respiration (after addition of ADP) by basal respiration. (**F**) Coupled respiratory control ratio (RCR) was calculated by dividing coupled respiration rates (after ADP) by OCR after oligomycin. (**G**) Maximal or uncoupled RCR was calculated by dividing maximal respiration rates (after FCCP) by OCR after oligomycin. Cr supplementation selectively increased coupling-associated parameters in isolated mitochondria from both female and male 3xTg mice. Student’s *t*-test or Mann-Whitney U (MWU); *n* = 5 wells/group; * *p* < 0.05; ** *p* < 0.01.

To assess whether the enhanced spatial learning with Cr supplementation in female 3xTg mice was also associated with increased hippocampal bioenergetics and whether 3xTg male mice demonstrated a different mitochondrial response to Cr supplementation that could account for their impaired performance, functional analyses were conducted in isolated mitochondria in a subset (*n* = 3 pairs of mice) of 3xTg mice after MWM testing (Figure 5). All OCR parameters were corrected for non-mitochondrial respiration (after blockade with antimycin and rotenone).

Across all functional mitochondrial experiments, the pattern of results was similar regardless of sex. No significant differences were found in baseline OCR in mitochondria from 3xTg mice as a function of diet (*p* = 0.1–0.7; Figure 5B). Coupled respiration, measured after the addition of oligomycin, was significantly enhanced with Cr supplementation (*p* < 0.05; *t*-test or MWU; Figure 5C). The rate of coupled respiration relative to baseline levels, considered a measure of coupling efficiency, was also enhanced with dietary Cr (*p* = 0.01–0.002; Figure 5E), as was the coupled RCR (*p* < 0.05; Figure 5F). Maximal respiration rates (after the addition of FCCP, an uncoupling agent) were not significantly different with Cr (*p* = 0.051–0.9; *t*-test or MWU; Figure 5D). The RCR associated with maximal respiration (e.g., uncoupled RCR) was not affected by diet (*p* = 0.8–0.9; *t*-test or MWU; Figure 5G). These data demonstrate that despite the sex-specific effects on spatial learning, Cr supplementation augmented aspects of mitochondrial function, particularly those related to the capacity for coupling ATP production to ETC activity, in both sexes in the 3xTg AD model.

### 3.5. Hippocampal Cr Levels

**Figure 6:**
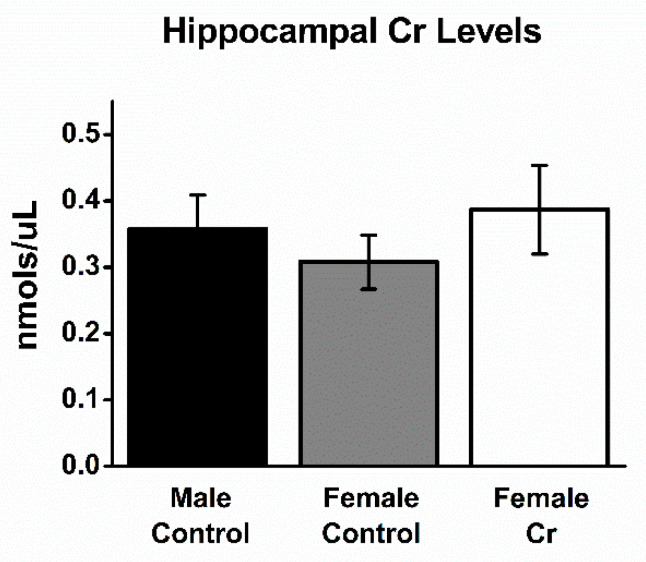
Levels of Cr in the hippocampus after 8 weeks of dietary intervention. Levels of Cr were measured in the hippocampi of Cr-supplemented female and control-fed male and female mice from tissue homogenates using a colorimetric kit (BioVision Inc.). ANOVA: female control: *n* = 3; female Cr: *n* = 5; male control: *n* = 3; *p* > 0.05.

After MWM training, Cr levels were measured in hippocampal homogenates in a subset of 3xTg mice. No significant differences were found between Cr levels among the three groups of 3xTg mice (male control: 0.358 ± 0.051; female control: 0.321 ± 0.088; female Cr: 0.387 ± 0.078 nmols/µL; ANOVA: F(2, 8) = 1.43, *p* = 0.3; Figure 6).

### 3.6. Western Blots

#### 3.6.1. Transcription Factors and Related Proteins

**Figure 7:**
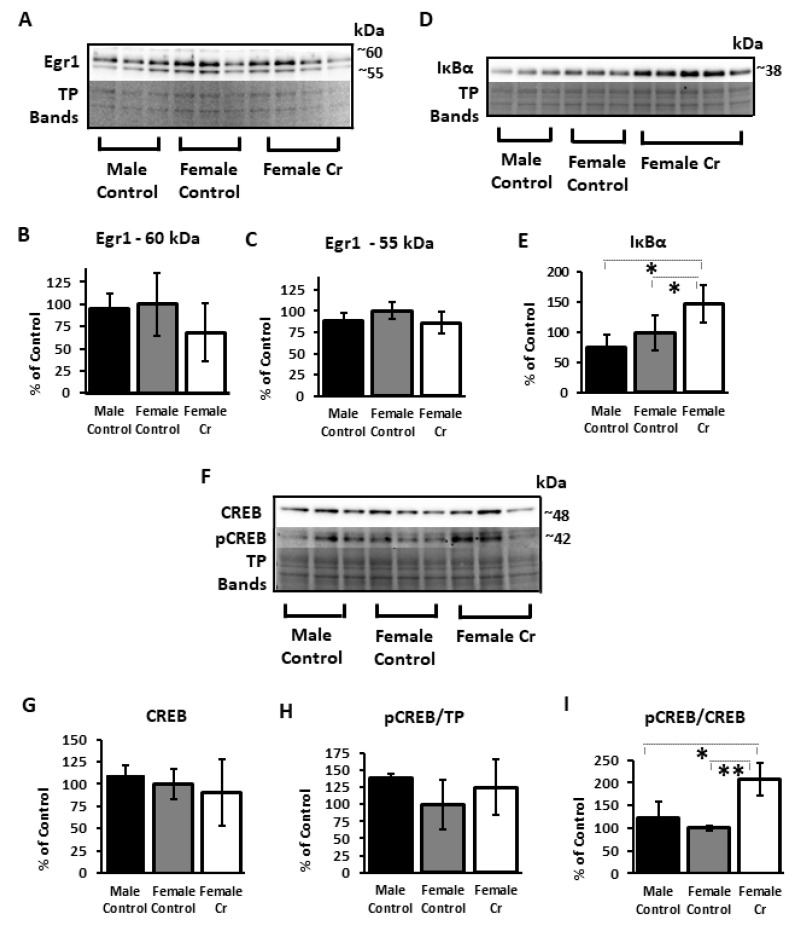
Relative hippocampal levels of transcription factors and related proteins in control and Cr-supplemented 3xTg mice. Representative Western blots detecting (**A**) early growth response 1 (Egr1), (**D**) inhibitor of nuclear factor kappa b, alpha (IκBα), (**F**) cAMP response element-binding protein (CREB), and phosphoCREB (pCREB). Total protein (TP) was detected with Ponceau S staining, with representative bands from total protein staining shown to indicate sample loading. Bar graphs depict densitometry values of (**B**) Egr1 at approximately 60kDa and (**C**) 55 kDa, as well as (**E**) IκBα, (**G**) CREB, and (**H**) pCREB after normalizing to total protein bands and expressed as the percentage change from control means (female control-fed; 100%) ± SD. (**I**) Relative levels of pCREB normalized to CREB. ANOVA or Brown–Forsythe: *n* = 3–5; * *p* < 0.05; ** *p* < 0.01.

To examine the presence of potential sex differences in typical 3xTg mice and to determine the effects of Cr supplementation on transcriptional regulation in females demonstrating enhanced cognitive performance, Western blots of several key transcription factor proteins implicated in learning and memory were conducted in hippocampal homogenates after MWM. Probing with anti-Egr1 antibody revealed two bands at ~55 and ~60 kDa, with no significant differences in densitometry values of either band after normalizing to total protein values (~55 kD: F(2, 7) = 1.19, *p* = 0.4; ~60 kDa: F(2, 2.57) = 0.35, *p* = 0.7; BF) (Figure 7A–C). In contrast, levels of IκB, an inhibitor of NF-κB activity, were significantly different (F(2, 5.67) = 12.39, *p* = 0.009; BF) and were dramatically increased in 3xTg females supplemented with Cr compared to those on the control diet (*p* = 0.03) (Figure 7D,E). Neither CREB (F(2, 8) = 0.47, *p* = 0.6) nor pCREB (F(2, 8) = 1.11, *p* = 0.4) were significantly different when normalized to total protein. However, levels of pCREB normalized to CREB were significantly different (F(2, 8) = 11.32, *p* = 0.009) and were more than doubled in Cr-supplemented 3xTg females versus their control-fed counterparts (*p* = 0.01) (Figure 7F–I; see Supplementary Figure S1 for additional images of Western blot membranes).

#### 3.6.2. Plasticity Proteins

**Figure 8:**
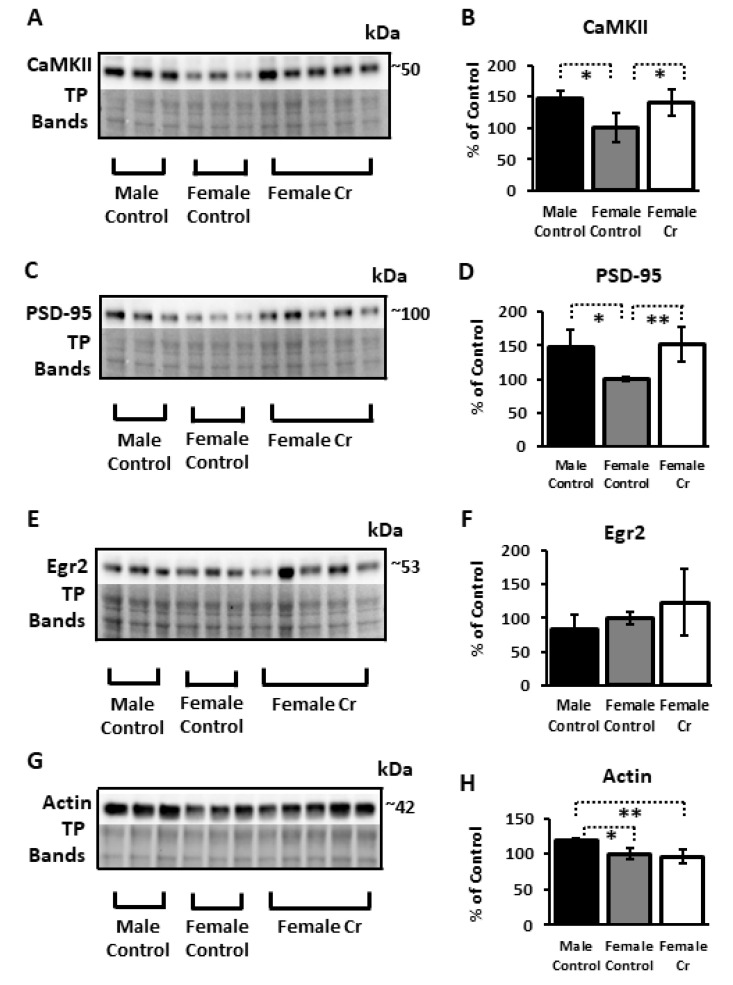
Relative hippocampal levels of nuclear factor kappa b (NF-κB)-associated plasticity proteins in control and Cr-supplemented 3xTg mice. Representative Western blots depicting protein bands and relative protein levels, respectively, as measured by densitometry after normalizing to total protein (TP; Ponceau S staining or Stain-Free^TM^ for actin) and expressed as the percentage change from female control-fed mice (100% ± SD) of (**A**,**B**) calcium-calmodulin-dependent protein kinase II (CaMKII), (**C**,**D**) postsynaptic density protein 95 (PSD-95), (**E**,**F**) early growth response protein 2 (Egr2), and (**G**,**H**) actin. ANOVA: *n* = 3–5; * *p* < 0.05; ** *p* < 0.01.

In addition to transcription factors implicated in cognition and experience-dependent plasticity, protein levels of several targets of these transcriptional regulators were examined. Significant sex differences were detected in both CaMKII and PSD-95 in 3xTg mice, with significant reductions in females relative to males (*p* = 0.019 and *p* = 0.036, respectively)—reductions that were abolished with Cr supplementation in females (*p* = 0.025 *p* = 0.007, respectively, compared to control-fed 3xTg females). Egr2, a downstream target of NF-κB in neurons (and a transcription factor itself) that has been implicated in learning and memory, was not significantly altered as a function of sex or diet (F(2, 8) = 1.16, *p* = 0.37). Actin, a key constituent of the cytoskeleton and regulator of dendritic spine dynamics, was significantly downregulated in 3xTg females compared to males (*p* = 0.01) and was not restored with Cr supplementation, where levels remained significantly reduced compared to males (*p* = 0.002) (Figure 8).

#### 3.6.3. Mitochondrial Proteins

**Figure 9:**
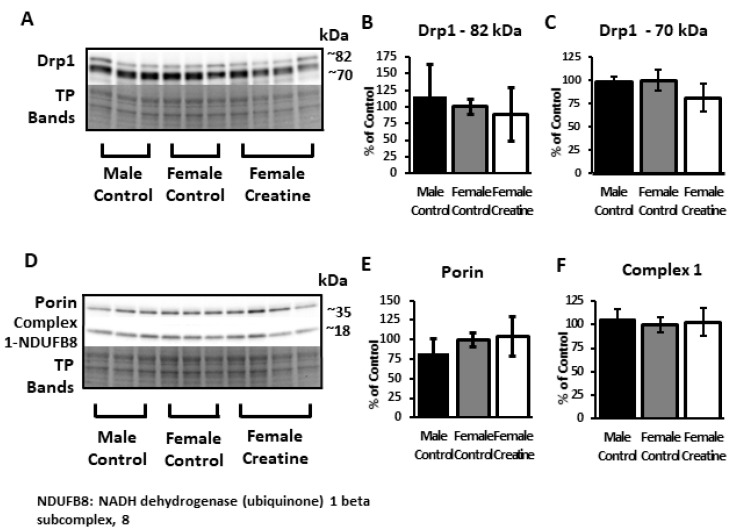
Relative hippocampal levels of mitochondrial proteins in control and Cr-supplemented 3xTg mice. Representative Western blots detecting (**A**) Drp1 (82 kDa and 70 kDa bands), (**D**) porin, and NDUFB8, a subunit of the electron-transport chain protein Complex I. Total protein (TP) was measured with Ponceau S staining (representative bands shown to indicate sample loading). Bar graphs (**B**,**C**,**E**,**F**) depict densitometry values after normalizing to total protein bands and are expressed as the percentage change from female control-fed means (100%) ± SD. ANOVA: *n* = 3–4.

Several mitochondrial-associated proteins were probed in an attempt to elucidate putative molecular underpinnings for the noted enhanced OCR with Cr supplementation in isolated mitochondria from both sexes. Western blots using anti-Drp1, a key regulator of mitochondrial fission [44], revealed two bands at ~72 and ~80 kDa, which were not significantly different between diets or sex (ANOVA; ~72 kDa: F(2, 7) = 0.4, *p* = 0.7; ~80 kDa: F(2, 8) = 2.78, *p* = 0.13). Similarly, no significant differences were detected in either porin (VDAC1) ((F(2, 7) = 0.18, *p* = 0.8), a constituent pore-forming protein of the outer mitochondrial membrane that permits subcellular ATP diffusion, or NDUFB8 complex I subunit of the ETC (F(2, 7) = 0.26, *p* = 0.8) (Figure 9). Although the Seahorse assay measures complex-I-dependent respiration, the antibody used to measure the complex I subunit also detects bands for subunits of complexes II–V. Analysis of densitometry values found no significant differences in levels of subunits to detect complexes II–V (*p* > 0.05 for all complexes; ANOVA, KW, and BF; see Supplementary Figure S1 for Western blot membranes).

#### 3.6.4. AD-Related Proteins

**Figure 10:**
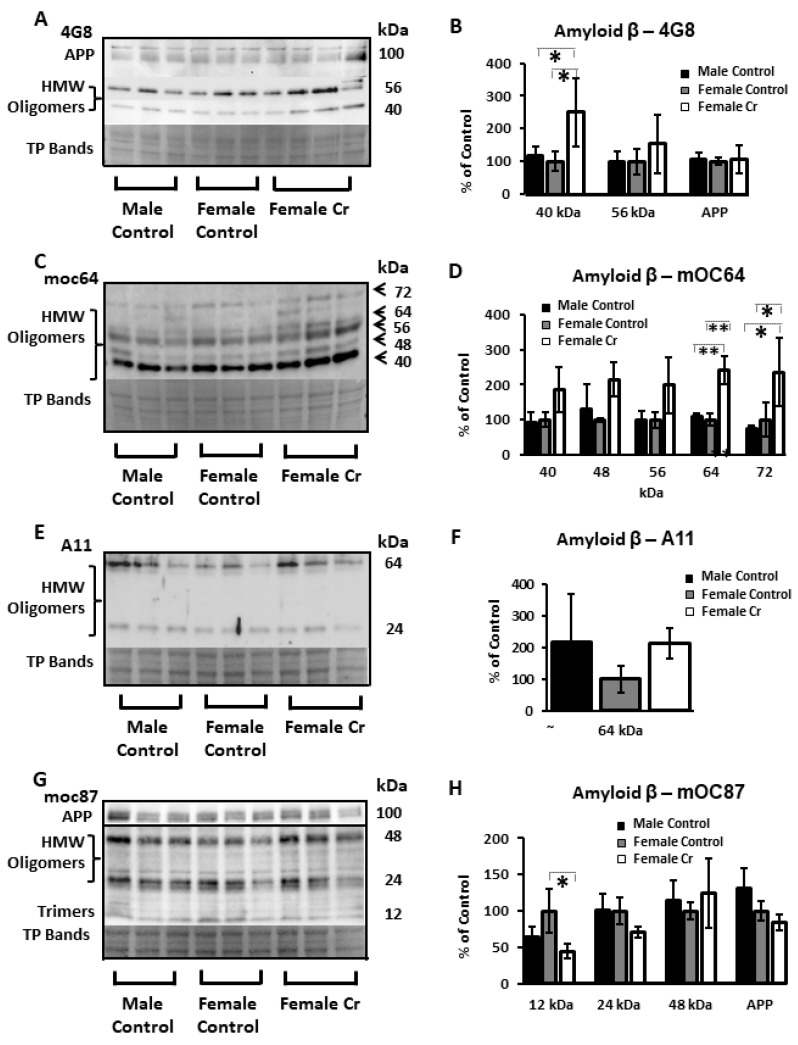
Relative hippocampal levels of amyloid β (Aβ) and amyloid precursor protein (APP) in control and Cr-supplemented 3xTg mice. Representative Western blots detecting Aβ (and APP in some cases) with the antibodies (**A**) 4G8, (**C**) moc64, (**E**) A11, and (**G**) moc87. Total protein (TP) was measured with Ponceau S staining (representative bands shown to indicate sample loading). Bar graphs (**B**,**D**,**F**,**H**) depict densitometry values after normalization to total protein bands and expressed as the percentage change from female control-fed means (100%) ± SD. ANOVA or Kruskal–Wallis; *n* = 3–4. * *p* < 0.05; ** *p* < 0.01.

As key components of neuropathology in AD, levels of Aβ and hyperphosphorylated tau (a major driver of NFT), or “ptau”, were semi-quantified in hippocampal homogenates in 3xTg male and female mice as well as 3xTg females supplemented with Cr. Western blot experiments were done using several antibodies against Aβ, as studies have indicated condition-, epitope-, and conformation-specific expression of Aβ in immunoblotting experiments across a variety of antibodies [45]. Using the 4G8 antibody, which recognized several high-molecular weight Aβ oligomers at 40 and 56 kDa, as well as APP upstream of Aβ processing, indicated a significant increase of the 40 kDa species in females supplemented with Cr compared to both control-fed female (*p* = 0.027) and male (*p* = 0.43) mice. In contrast, analysis of densitometry using the antibody mOC64, which detected five bands ranging from 40 to 72 kDa, also indicated significant increases with Cr supplementation in 3xTg females, but at the highest molecular weight detected with this antibody (64 kDa: *p* = 0.001; 72 kDa: *p* = 0.038). The oligomeric-specific A11 antibody, which recognizes high-molecular weight oligomers but not monomers or fibrils, recognized two bands at 24 and 64 kDa in samples in the current study, which were not significantly different. Another antibody that recognizes APP, mOC 87, was also used and detected high-molecular weights oligomers (24 and 48 kDa), as well as bands consistent with trimers at 12 kDa. Of the four bands measured, only the 12 kDa band was significantly altered. Here, Cr supplementation significantly reduced the levels of Aβ trimers (*p* = 0.017), unlike the increases seen with Cr supplementation at higher molecular weights using other antibodies (Figure 10; see Supplementary Figure S2 for Western blot membranes).

**Figure 11:**
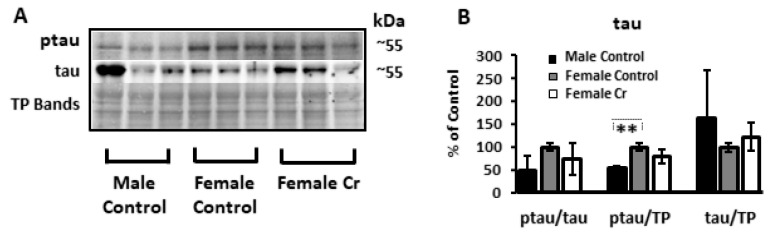
Relative hippocampal levels of hyperphosphorylated tau protein in control and Cr-supplemented 3xTg mice. (**A**) Representative Western blots detecting hyperphosphorylated tau (ptau) and total tau. Total protein (TP) was measured with Ponceau S staining (representative bands shown to indicate sample loading. (**B**) Bar graph depicts densitometry values after normalization to total tau and to total protein (TP) bands, which are expressed as the percentage change from female control-fed means (100%) ± SD. ANOVA or Brown–Forsythe: *n* = 3. ** *p* < 0.01.

With respect to ptau, the levels were not affected by Cr supplementation in females (when normalized to total tau or total protein). However, sex differences were detected (with LSD post hoc tests) in the amount of ptau relative to total hippocampal protein (*p* < 0.01), indicating elevated levels of pathological tau in the hippocampus in 3xTg female mice relative to males (Figure 11). The levels of total tau normalized to total protein were not significantly different overall (F(2, 2.38) = 0.74, *p* = 0.6; BF).

### 3.7. Correlational Analysis with Cr, Memory, and Protein Levels

**Figure 12:**
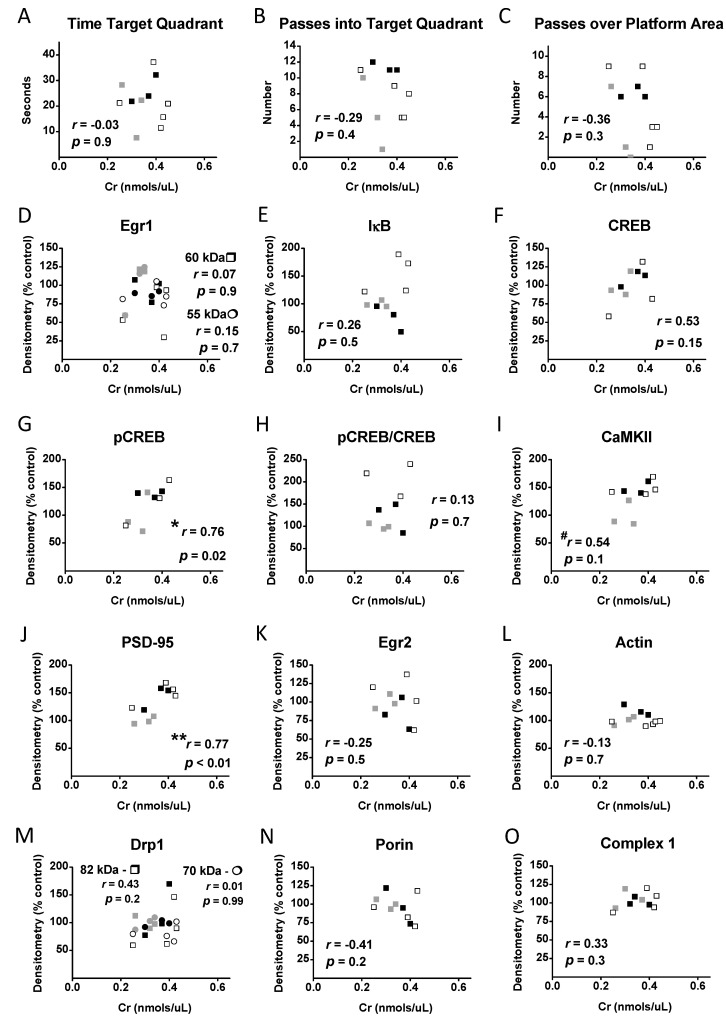
Correlational analyses between Cr, memory, and protein levels. Pearson correlation coefficients between hippocampal Cr levels and (**A**–**C**) MWM probe trial parameters and relative normalized densitometry values from Western blot experiments measuring (**D**–**H**) transcription factor, (**I**–**L**) plasticity-related, and (**M**–**O**) mitochondrial proteins in control-fed male (black squares) and female (gray squares) 3xTg mice, as well as female 3xTg mice on a Cr-supplemented (white squares) diet. Note: *n* = 3–5, ^#^ *p* = 0.1, * *p* < 0.05; ** *p* < 0.01.

In order to investigate the potential relationships between hippocampal Cr levels and spatial memory, as well as protein levels of transcription, plasticity, and mitochondrial proteins, Pearson correlation coefficients were computed between each of the parameters measured in the probe (memory) trial and Cr levels, as well as between densitometry values and Cr levels. No significant correlations were found between Cr levels and any of the parameters used to assess memory in the MWM. Levels of pCREB (normalized to total protein) were significantly positively correlated with Cr levels in the hippocampus in 3xTg mice (*r* = 0.76, *p* = 0.02), as were levels of PSD-95 (*r* = 0.77, *p* < 0.01). Although the correlation coefficient for the relationship between CaMKII levels and Cr was considered moderate (*r* = 0.54), this value only approached significance (*p* = 0.1) (see Figure 12).

**Figure 13:**
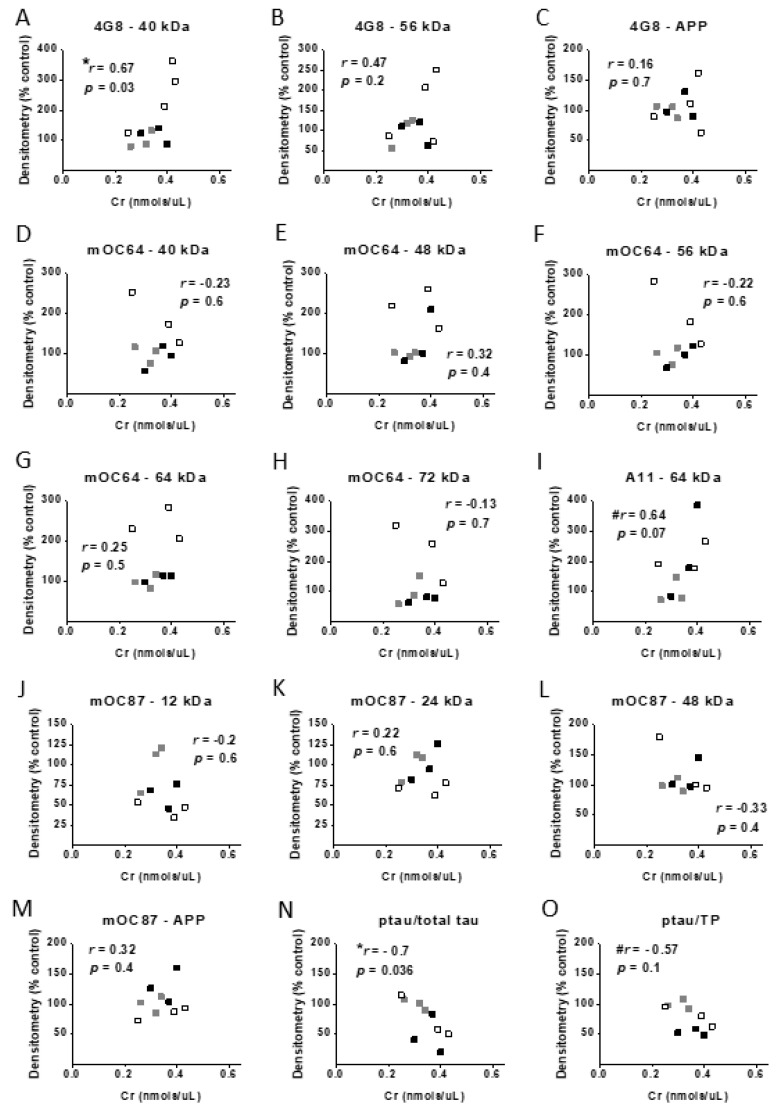
Correlational analyses between Cr and Alzheimer’s disease- (AD) related protein levels. Pearson correlation coefficients between hippocampal Cr levels and relative normalized densitometry values from Western blot experiments measuring amyloid β (Aβ) species and/or APP with antibodies (**A**–**C**) 4G8, (**D**–**H**) moc64, (**I**) A11, and (**J**–**M**) moc87 or (**N**,**O**) AT8 to measure hyperphosphorylated tau in control-fed male (black squares) and female (gray squares) 3xTg mice, as well as female 3xTg mice on a Cr-supplemented (white squares) diet. Note: *n* = 3–4, * *p* < 0.05, ^#^ *p* < 0.01.

Correlation coefficients were also calculated between hippocampal Cr levels and densitometry values that were semi-quantified for Aβ and tau (ptau and total tau) to further investigate relationships between brain Cr levels and molecular hallmarks of AD pathology. Levels of Aβ detected by 4G8 at 40 kDa were significantly positively correlated with hippocampal Cr (*r* = 0.67, *p* = 0.03). Densitometry values of the 64-kDa band using the A11 antibody were marginally significantly correlated with Cr levels (*r* = 0.64, *p* = 0.07). In contrast to the positive significant correlation with Aβ, Cr levels were significantly negatively correlated with ptau normalized to total tau (*r* = 0.7, *p* < 0.05) and marginally significantly correlated with ptau normalized to total protein (*r* = −0.57, *p* = 0.1) (see Figure 13). A moderate correlation between total tau and Cr levels was found, which did not reach statistical significance (*p* = 0.1). Overall, such data further implicate brain Cr as a mediating factor in the expression of AD-relevant biomolecules.

To further our understanding of the relationships between AD-related neuropathology and memory, correlations were also computed between densitometry values for Aβ, ptau, and total tau in 3xTg mice and memory parameters in the present study. Only Aβ trimers at 12 kDa were associated with memory, as indicated by a significant negative correlation with the number of passes over the platform area (*r* = −0.82, *p* = 0.007) and a marginally significant negative correlation with entries into the target quadrant (*r* = −0.63, *p* = 0.07).

## 4. Discussion

**Figure 14:**
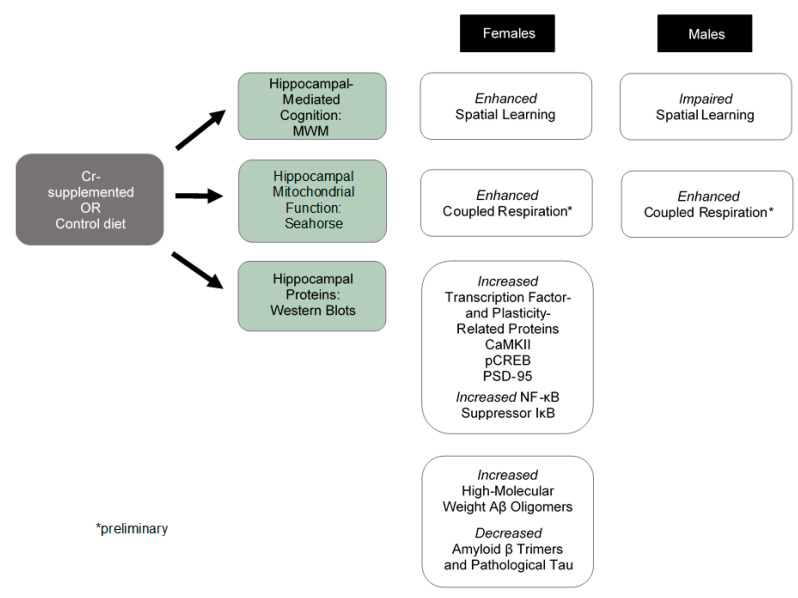
Summary of findings in 3xTg mice. Cr supplementation resulted in sex-specific effects on spatial cognition, as measured by the Morris water maze (MWM). Preliminary findings in isolated mitochondria from both sexes demonstrated consistent effects of Cr supplementation on coupled respiration. Western blot experiments were conducted in female 3xTg mice only due to the small sample size of Cr-fed males.

The present study reports on the effects of dietary Cr supplementation on multiple parameters in the 3xTg mouse model of AD, including spatial learning and memory, mitochondrial function, as well as levels of transcription factors and proteins implicated in learning, memory, and mitochondrial activity. Importantly, sex differences were noted in the effects of Cr supplementation on spatial cognition that could not likely be accounted for by corresponding sex differences in the effects of Cr supplementation on mitochondrial respiration, for which enhancements were seen in isolated hippocampal mitochondria from both sexes in preliminary experiments (see Figure 14 for a summary of findings).

Under normal dietary conditions, male 3xTg mice outperformed females on the MWM, including the number of passes into the platform area, indicating more severe memory impairment in female 3xTg mice relative to males, consistent with other studies in this AD model [36]. Although there was no significant difference between females as a function of diet, Cr supplementation abolished these sex differences, as Cr-supplemented females performed at levels on par with control-fed 3xTg males in the MWM. Moreover, spatial learning was enhanced with Cr in female 3xTg mice, as demonstrated by a decreased escape latency and a significantly greater reliance on spatial search strategies by the end of training, which was considered indicative of forming a spatial map [41]. In contrast, male 3xTg mice supplemented with Cr used spatial strategies significantly less than their control-fed counterparts and instead relied primarily on repetitive looping by the end of training, a strategy that is typically seen early on during training (e.g., acquisition phase).

One plausible reason that a more robust overall effect of Cr supplementation on cognition was not observed in females may be due to the age used (7 months) and duration of supplementation (8 weeks). Both mitochondrial deficits [46] and synaptic deficits [47] are present by 6 months of age in 3xTg mice, with mitochondrial alterations occurring even earlier in females [46]. Thus, supplementation with Cr prior to the expression of these deficits may have potentiated its cognitive-enhancing effects in female 3xTg mice. More robust effects across cognitive parameters may have also been detected with larger sample sizes than used here, which may be considered smaller than normal for MWM experiments. For example, although the search strategy analysis yielded statistical differences with Cr in males, escape latency was only marginally significant in this group with Cr supplementation, an effect that may have reached significance with a larger sample size. It is worth noting, however, that the calculated effects sizes of Cr supplementation on escape latency in 3xTg females and males (Cohen’s *d* = 1.7 and 1.4, respectively) are considered large [48], resulting in smaller sample sizes being required for adequate statistical power.

Given the well-known effects of Cr supplementation on muscle biology, it could be argued that the enhanced performance in the motor-dependent MWM seen in Cr-fed 3xTg female mice may be secondary to muscle-enhancing effects of Cr consumption. However, several additional findings of the present study argue against such an interpretation, including indicators of impaired performance in the MWM in male 3xTg mice, as well as a lack of significant differences in swim speed in Cr-fed mice. Additionally, analysis of spatial strategy, which could be considered less reliant on the motor capabilities of the animals than other parameters (e.g., escape latency, whereby slower animals would take longer to find the platform) indicated diet-specific differences in the degree of reliance on search strategies that were in line with performance in the acquisition (e.g., learning) phase and the probe trial (e.g., memory phase).

Interestingly, over the course of the study, food intake was significantly higher in female 3xTg mice relative to males, regardless of dietary intervention. To our knowledge, this is the first report of differences in food intake in 3xTg mice as a function of sex and adds to the growing list of sex differences reported in this AD model, including neuropathology, spatial memory [36], and olfactory processing [49], to name a few. Interestingly, abnormal appetite and eating behaviors have been shown to occur more frequently in women with mild AD (similar to the state of disease progression in 3xTg mice in the present study) than in men [50]. Overexpression of tau in mice has been shown to increase appetite and food consumption [51], although no sex differences were detected. Increased food intake in 3xTg females would correspond to increased Cr consumption relative to 3xTg males, however, it could not account for the deficits in spatial cognition noted in male 3xTg mice supplemented with Cr, and therefore could not explain the sex effects of Cr supplementation reported here.

Other studies have demonstrated similar sex-dependent therapeutic effects of oral Cr, including reductions in depressive-like symptoms in female rats yet increases in males in the forced swim test, which is used as a mouse model of depressive behavior in humans [38], whereas such symptoms were increased in male rats with oral Cr. These data suggest antidepressive effects of Cr in the female mammalian brain. Although the authors logically speculate that the opposing therapeutic effects of Cr may be related to differences in sex hormones and sex differences in Cr metabolism, one recent study reporting clear and contrasting sex-dependent molecular changes in the brains of men and women with depression [52] suggests that the sex-dependent antidepressant effects of Cr may not be due to inherent sex differences related to Cr metabolism but rather to Cr altering the molecular profile in the brain that is sexually divergent in the basal condition. Thus, the results presented here may be due to inherent sex differences in 3xTg mice rather than sex differences attributed to the brain Cr system, per se. Although research in non-diseased rodents so far indicates enhanced cognition in both males [19] and females [53], further research examining the effects of dietary Cr between males and females in the typical brain is required to confirm or refute whether sex-specific effects of Cr in pathological states are inherent to sex differences in Cr regulation and metabolism or to disease-specific sex differences.

In healthy control mice, we previously found enhanced MWM performance accompanied by increased hippocampal OCR that was specific to parameters measuring coupled respiration and capacity with dietary Cr [19], similar to that seen in female 3xTg mice after Cr supplementation. Surprisingly, however, isolated mitochondria from male 3xTg mice that demonstrated Cr-induced cognitive impairment also exhibited enhanced OCR, downplaying the contribution of mitochondrial enhancement to the noted cognitive enhancements that have been repeatedly reported with Cr supplementation. The results presented herein, however, may be considered preliminary in light of the limited number of functional mitochondrial assays performed across pairs of both male and female 3xTg mice as a function of dietary intervention.

Despite the noted enhancement in mitochondrial function, no significant differences were detected in any of the mitochondrial-related proteins examined with Cr supplementation in female 3xTg mice, including complex I, an effect we previously detected in C57BL/6 male mice on the same diet. Although levels of the mitochondrial fission protein Drp1 were also unaffected by Cr supplementation, this does not rule out changes to mitochondrial fission dynamics with Cr supplementation in the 3xTg model. For example, studies in AD models have shown that increased phosphorylation and S-nitrosylation of Drp1 could affect mitochondrial fission by promoting the translocation of Drp1 to the mitochondria in the absence of increased levels of Drp1 [54,55]. Since the uncoupler-induced OCR was not different between the Cr and control-fed mice, no statistically significant differences in the levels of ETC complexes I, III, or IV proteins were expected. However, since there was a statistically significant difference in ADP-stimulated respiration, there may be a deficiency in ADP transport or phosphorylation. Future work is needed to more fully investigate the molecular mechanisms associated with the preliminary results of enhanced coupled respiration in 3xTg mice of both sexes.

The present study found a robust upregulation of the inhibitory component of NF-κB, IκB, in 3xTg females supplemented with Cr, consistent with reduced NF-κB activity. This finding is in contrast to the downregulation of this inhibitory component (consistent with upregulation of NF-κB activity) previously found in healthy C57BL/6 male mice [19]. Inhibition of NF-κB has been shown to inhibit structural changes associated with plasticity in the hippocampus, including neural growth and dendritic branching [56]. Downregulation of NF-κB in neurons has also been shown to decrease CREB activation via protein kinase A [57], which phosphorylates CREB at serine 133 [58]. These effects are in contrast to the increased phosphorylation of CREB in Cr-supplemented 3xTg females that exhibited IκB upregulation. A decrease in NF-κB transcriptional activity, however, in the presence of increased CREB activation, as found here with Cr supplementation in 3xTg females, is also consistent with literature indicating that CREB and NF-κB compete for similar binding sites of the co-activator CREB binding protein within the nucleus [59]. Additionally, both CREB and NF-κB exert transcription influence on several shared downstream targets, including PSD-95 [23], and share common activators, including CaMKII [21,60]. In C57BL/6 male mice, CREB was not affected by Cr supplementation, as has been demonstrated with Cr administration directly into the hippocampi of male rats [61]. Importantly, levels of pCREB/CREB are significantly reduced in the hippocampus of 3xTg mice [36]. Although our studies in both 3xTg and control mice suggest that Cr supplementation may upregulate PSD-95, possibly through distinct pathways in the pathological and typical mammalian brain (with evidence indicating CaMKII/NF-κB/PSD-95 in healthy conditions and CaMKII/CREB/PSD-95 in AD-like conditions), we cannot rule out sex-dependent effects on these molecular pathways implicated in synaptic plasticity and memory in the hippocampus as a contributing factor as well, given our lack of data on the effects of Cr supplementation in typical female mice.

The present results in the 3xTg model also suggest potential anti-inflammatory effects of Cr, as NF-κB is a potent inflammatory regulator with diverse effects upon activation in neurons (e.g., neuroprotective effects) versus glia (e.g., inflammatory effects) [62]. Previous studies have reported anti-inflammatory effects of Cr in vitro [63]. It is important to consider, however, that data collected in the present study were from hippocampal homogenates that would include protein from both glia and neurons, both of which express this inhibitory portion of the NF-κB complex, thereby ruling out an ability to investigate the effects of Cr supplementation on IκB and NF-κB activation on a cellular level. A decrease in IκB in homogenates is, therefore, not incompatible with an increase the activity of NF-κB in neurons, as reported in C57BL/6 male mice. Additional studies are required to examine the effects of Cr supplementation with greater subcellular resolution in the brain.

Several species of Aβ revealed with various antibodies, notably high-molecular weight oligomers, were increased with Cr supplementation. The 40 kDa high-molecular weight oligomer recognized by the 4G8 antibody was both significantly increased with oral Cr supplementation and significantly positively correlated with hippocampal Cr levels, providing converging evidence of the effects of Cr on this specific Aβ species. No significant differences were detected using A11, the conformation-specific antibody shown to recognize oligomers and not monomers or fibrils (aka “prefibrillar”). Moreover, no significant differences were detected in levels of APP (confirmed with two antibodies), indicating that noted Cr-induced alterations to Aβ levels were due to changes in amyloidogenic processing and not to the bioavailability of its precursor protein. Of the various Aβ species probed, only the 12 kDa trimer detected with the mOC87 antibody that recognizes fibrils was significantly lower in Cr-fed female 3xTg mice. The pattern of molecular amyloidogenic changes suggests a complex interaction with brain Cr and Aβ, whereby Cr supplementation may specifically downregulate low-molecular weight Aβ trimers whilst upregulating specific high-molecular weight oligomers or fibrils. Notably, in addition to its downregulation with Cr supplementation, only the 12 kDa mOC87-detected band was significantly associated with memory performance in the MWM, with a strong negative correlation between Aβ trimers and the number of passes over the platform area. Importantly, studies have demonstrated neurotoxic effects specifically of Aβ trimers, including cell death [64] and impaired hippocampal synaptic plasticity [65]. Consistent with findings reported here, cognitive impairment was spared with reduced levels of Aβ trimers in the hippocampus of APP/PSS1 mouse, another AD model [66]. Together, these data provide converging evidence of the importance of this Aβ species in spatial cognition and the therapeutic effects of their reduction in the context of AD.

In addition to Aβ trimers, other studies have found significant associations between AD-related biomolecules and cognition. For example, in the Tg2576 AD mouse model, which exhibited Aβ accumulation but did not develop NFT, a significant negative correlation between forebrain levels of large 40 and 56 kDa oligomeric assemblies and spatial memory in the MWM was noted [67]. We detected a similar band in our Western blot experiments that was not significantly altered with Cr supplementation (using two different antibodies), the levels of which were also highly variable, especially across female 3xTg mice in particular. Others have reported a significant positive correlation between hippocampal levels of Aβ42 and spatial working memory deficits in the 3xTg model [68], although putative relationships between tau-related pathology and cognition were not examined in this study. Although tau-related pathology was not associated with cognition in the present study, hippocampal Cr levels were negatively correlated with levels of hyperphosphorylated tau. Park et al. [69] found that tau binds to several energy-regulating proteins in brain tissue and demonstrated a strong affinity for tau to CrK in particular, as it accounted for over 50% of detected proteins in co-immunoprecipitation experiments. Few studies, however, have examined the relationship between tau signaling and the creatine energy system, either under physiological conditions or in the context of AD, where evidence indicates perturbations in both mechanisms. Our data, thus, indicate a relationship between brain Cr levels and tau signaling in the AD-like brain that warrants further investigation.

In this study, traditional neuropathological characteristics of AD in response to Cr supplementation were not measured due to findings showing limited hippocampal Aβ plaque deposition or NFT (associated with tau hyperphosphorylation) at the age of mice used in the present study, features which are not robust in the hippocampus until ~12 months of age [47,70]. Therefore, we evaluated key AD-related proteins in response to Cr supplementation at the molecular level. Cognitive deficits, however, develop earlier than overt neuropathology in the 3xTg model, including spatial learning and memory deficits, which can be detected at around 6 months of age [35,71]. The age of mice chosen for this study, therefore, coincided with an age that cognitive deficits would be expected and before significant neuropathology occurs. Although 3xTg females demonstrated impaired spatial cognition relative to males, no sex differences were found in hippocampal Aβ levels in the present study, whereas total levels of hyperphosphorylated tau were significantly higher in female 3xTg mice relative to males. These findings are consistent with previous studies indicating impaired hippocampal-mediated cognition [70], including performance in the MWM [36], as well as elevated tau pathology in females in this strain [34]. In contrast to our findings, greater hippocampal Aβ plaque burden has been previously reported in 3xTg females compared to males [36,70].

Notably, levels of key plasticity-associated proteins, including CaMKII, PSD-95, and actin, were downregulated in females relative to male 3xTg mice, with CaMKII and PSD-95 levels potentiated with Cr supplementation in females, which may have contributed to the improved performance in transgenic females with supplementation. In hippocampal neurons, 12-h treatment with Cr increased PSD-95 density, spine density, and mitochondrial respiration, similar to that reported here with in vivo Cr supplementation [72]. These effects were also demonstrated with neuronal overexpression of Drp1, unlike our findings in vivo. These authors argued that mitochondria biogenesis is both required for and a limiting factor in synapse formation and maintenance. As a key constituent of the postsynaptic density in dendritic spines, upregulation of PSD-95 is considered indicative of synaptogenesis and spine stability [73]. Actin, however, another well-known regulator of spine dynamics in neurons that is intimately involved in activity-dependent spine retraction and formation [74], was not altered with Cr supplementation in 3xTg females (or C57BL/6 in our previous study), indicating preferential effects of Cr on plasticity-associated proteins within the hippocampus rather than a generalized potentiating effect of mitochondrial biogenesis on synaptic proteins.

From a preclinical perspective, the regulation of CaMKII, PSD-95, and CREB phosphorylation with Cr supplementation in female 3xTg mice is particularly important in light of findings demonstrating their dysregulation in the AD post mortem brain [75,76,77]. Further, repeated failures of current approaches to treat AD have spurred interest in those that target the synapse, including those that modulate CREB specifically [78]. The increase in CREB activation and the enhancements in hippocampal-related spatial cognition with Cr supplementation in females in this model, therefore, support its potential as a sex-specific therapy in AD.

In sharp contrast to its effects in female transgenic mice, Cr supplementation significantly impaired hippocampal-mediated spatial cognition in 3xTg males, as measured by the MWM. To our knowledge, this is the first study to report impaired cognition with Cr in vivo in either human or animal studies. Unfortunately, we cannot speculate on putative physiological underpinnings of this detrimental effect due to the small sample size in this group. Given that we have previously shown enhanced mitochondrial function with Cr supplementation in typical mice, we examined oxidative phosphorylation in hippocampal mitochondrial respiration in both sexes in the 3xTg model and found enhanced coupled respiration irrespective of sex, similar to our previous study in control mice. Thus, deficits in spatial cognition with Cr supplementation in 3xTg males do not appear due to a differential effect of Cr on mitochondrial function in this group. Our data overall point to suppressed plasticity in the hippocampus in 3xTg females that is accompanied by impaired hippocampal-mediated spatial cognition, providing evidence that Cr supplementation can restore synaptic protein levels, suppress hippocampal Aβ trimers, and alleviate cognitive deficits in females in the 3xTg model.

## 5. Conclusions

The present study investigated the behavioral, molecular, and bioenergetics effects of Cr supplementation in 3xTg mice, a common AD model, with a focus on the hippocampus, a region particularly affected in AD. This is the first to report sex-specific effects of Cr supplementation on spatial cognition in an AD model with perturbations in both Aβ and tau processing, with enhancements specifically in females that were accompanied by upregulations in several plasticity-related proteins, as well as differential effects on AD-related proteins. Despite the disparate behavioral response to Cr supplementation, preliminary data suggest that bioenergetic function is enhanced in mitochondria irrespective of sex in the 3xTg model (see Supplementary Figure S5 for a graphical summary of the study findings). Further studies are needed to address sex-dependent cognitive impairments associated with Cr supplementation in the AD-like brain and may be critically important for men at an elevated risk for AD, including those with a familial history of the disease or those with mild cognitive impairment, which is considered its prodrome. The results presented here suggest that Cr supplementation may represent an effective therapeutic approach for women with the disease or at elevated risk. Further studies are needed to evaluate the longer-term effects of Cr supplementation in this model and in older mice to determine its effects once AD-related neuropathology is more robust.

## Figures and Tables

**Table 1:** Primary antibodies used in Western blot experiments in 3xTg mice.

Antibody	Description	Supplier (Catalogue No.)	Dilution
Egr1	Rabbit polyclonal	Santa Cruz (sc-189)	1:2000
Egr2	Rabbit monoclonal	Abcam (ab108399)	1:7500
PSD-95	Rabbit monoclonal	Abcam (ab76115)	1:2000
IκBα	Rabbit monoclonal	Abcam (ab32518)	1:1000
CaMKII	Rabbit polyclonal	Santa Cruz (M-176)	1:2000
Drp1	Mouse monoclonal	Abcam (ab156951)	1:1000
Actin	Rabbit polyclonal	Sigma (A5060)	1:1000
Total OXPHOS rodent WB antibody cocktail	Mouse monoclonal	Abcam (ab110413)	1:1000
Porin	Mouse monoclonal	Abcam (ab14734)	1:1000
pCREB (at Serine 133)	Rabbit monoclonal	Abcam (ab32096)	1:1000
CREB	Rabbit monoclonal	Abcam (ab32515)	1:1000
AT8 (ptau at Ser202 and Thr 205)	Mouse monoclonal	Thermo Fisher Scientific (MN1020)	1:500
Total tau	Rabbit polyclonal	Abcam (ab39524)	1:1000
mOC87 (amyloid fibril & APP)	Rabbit monoclonal	Abcam (ab201062)	1:1000
4G8 (amyloid β 1–42)	Mouse monoclonal	Biolegend (800701)	1:1000
mOC64 (amyloid β 1–42)	Rabbit monoclonal	Abcam (ab201060)	1:1000
A11 (oligomeric amyloid β 1–42)	Rabbit polyclonal	Thermo Fisher Scientific (AHB0052)	1:1000

Abrreviations: Egr1 = early growth response protein 1; Egr2 = early growth response protein 2; PSD-95 = postsynaptic density protein 95; IκBα = inhibitor of nuclear factor kappa b, alpha; CaMKII = calcium-calmodulin-dependent protein kinase II; Drp1 = dynamin-related protein 1; OXPHOS = oxidative phosphorylation; CREB = cAMP response element-binding protein; pCREB = phosphoCREB.

## Supplementary Materials

The following are available online at https://www.mdpi.com/2072-6643/12/11/3589/s1: Figure S1: Western blot membranes for detection of transcription factor, plasticity, and mitochondrial proteins in the 3xTg hippocampus, Figure S2: Western blot membranes for detection of AD-related proteins in the 3xTg hippocampus, Figure S3: Correlational analyses between Cr, memory, and protein levels, Figure S4: Correlational analyses between Cr and AD-related protein levels, Figure S5: Summary of findings in 3xTg mice.
