# Supplementary material for: Sex-Specific Effects of Chronic Creatine Supplementation on Hippocampal-Mediated Spatial Cognition in the 3xTg Mouse Model of Alzheimer’s Disease
Source: Nutrients. 2020 Nov 23;12(11):3589. doi: 10.3390/nu12113589 (PMC7700653; doi:10.3390/nu12113589)
Supplement: Supplementary file 1 [file nutrients-12-03589-s001.zip › Snow et al Creatine 3xTg mice Nutrients - Supp Figures Rev.docx]

**Supplementary Figure 1**. **Western blot membranes for detection of transcription factor, plasticity, and mitochondrial proteins in 3xTg hippocampus.** Western blot membranes of hippocampal homogenates from 3xTg mice with antibodies against: (A) Egr1, (B) IκBα, (C) CREB, (D) pCREB; (E) CaMKII, (F) PSD-95, (G) Egr2, (H) Actin; (I) Drp1, (J) Porin, and (K) ETC Complexes I – V.*represents bands unrelated to stated protein detected in cases where the membrane was cut (dashed line) or stripped (≠) and incubated with a second antibody. All antibodies used were tested initially in independent membranes to confirm specificity of bands.


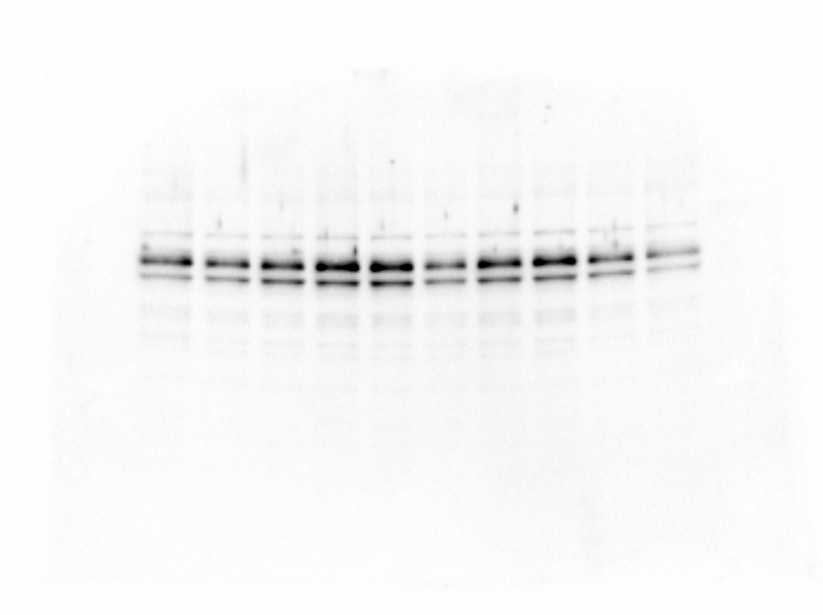

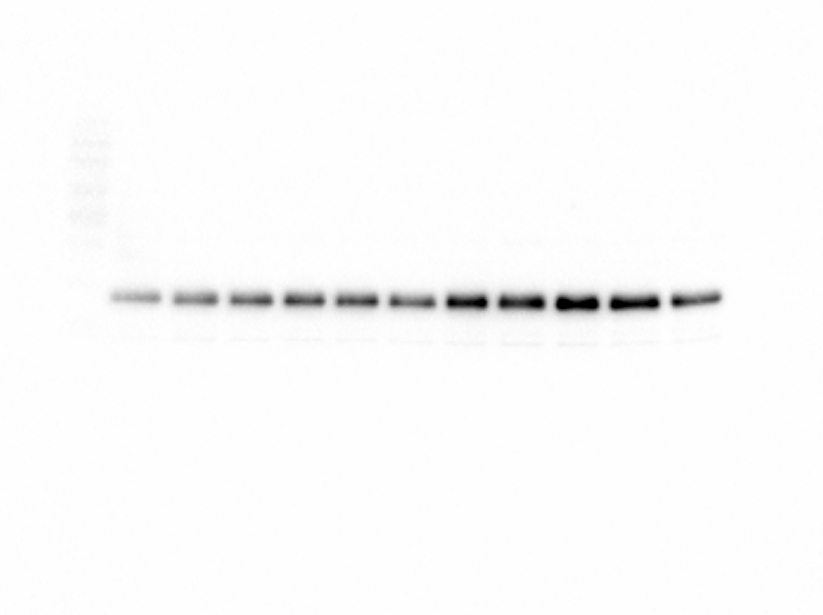
(A) Egr1 (B) IκBα

M M M F F F F F F F F

CO CO CO CO CO CO CR CR CR CR CR

M M M F F F F F F F

CO CO CO CO CO CO CR CR CR CR

~60 kDa

~55 kDa

~38 kDa

(C) pCREB (D) CREB


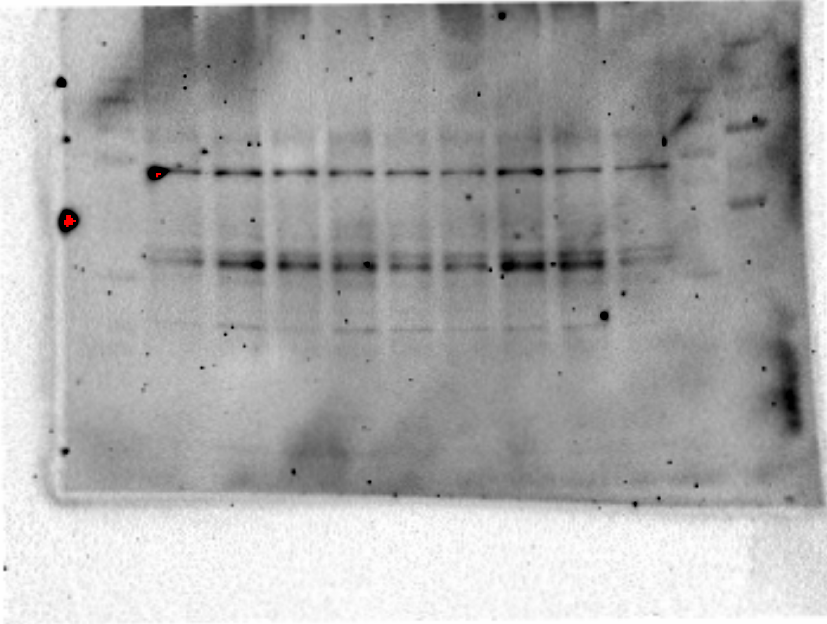

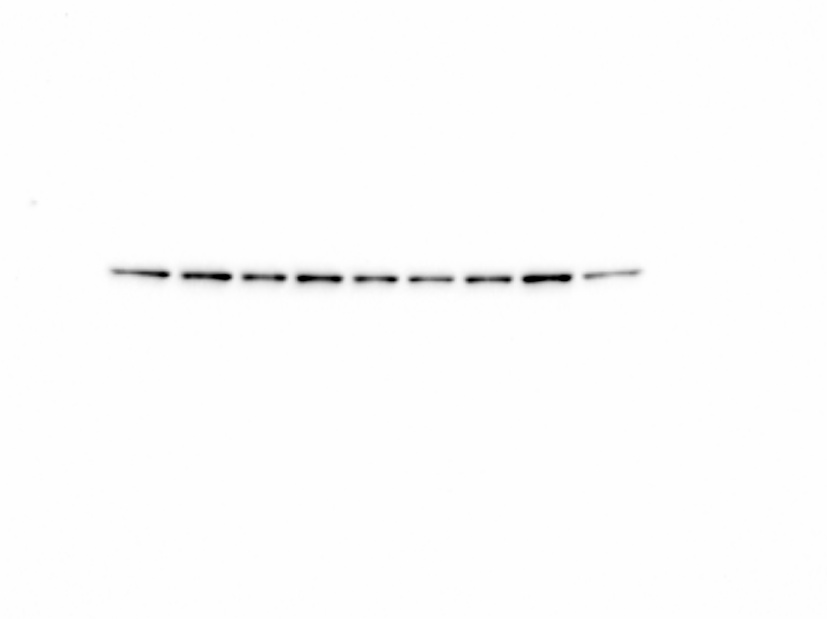


M M M F F F F F F

CO CO CO CO CO CO CR CR CR

M M M F F F F F F

CO CO CO CO CO CO CR CR CR

~48 kDa

~42 kDa


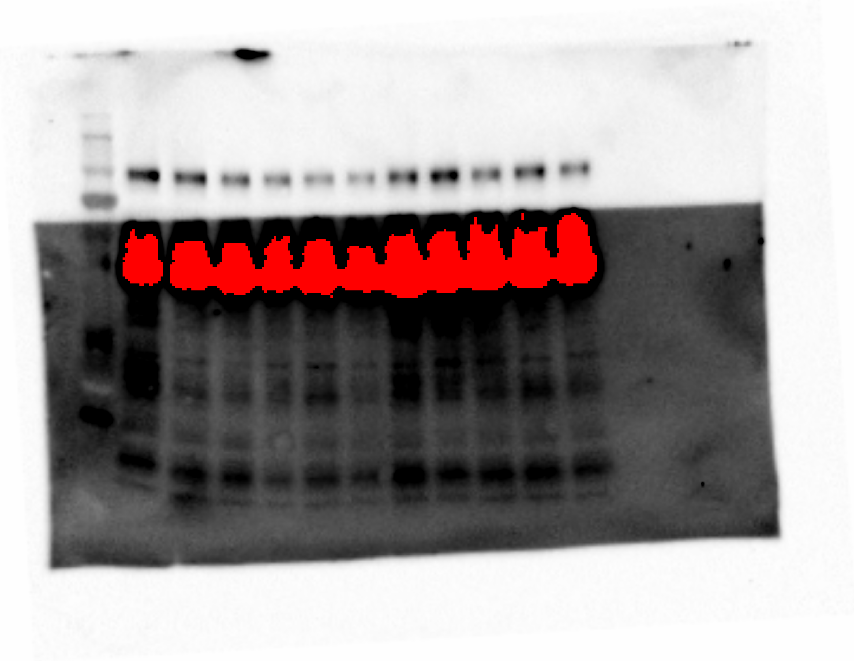
(E) CaMKII* (F) PSD-95*


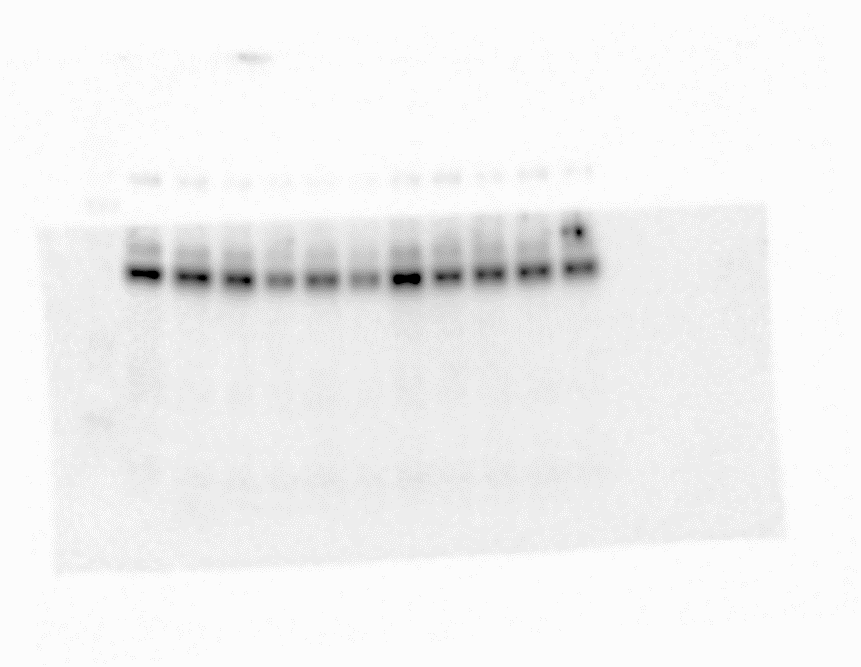


M M M F F F F F F F F

CO CO CO CO CO CO CR CR CR CR CR

M M M F F F F F F F F

CO CO CO CO CO CO CR CR CR CR CR

~100 kDa

~50 kDa


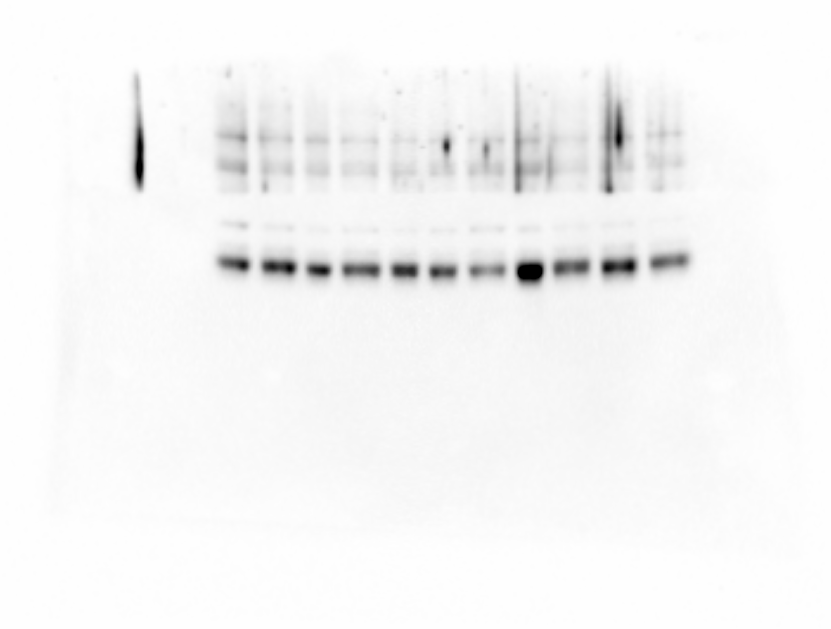
(G) Egr2 (H) Actin*

M M M F F F F F F F F

CO CO CO CO CO CO CR CR CR CR CR


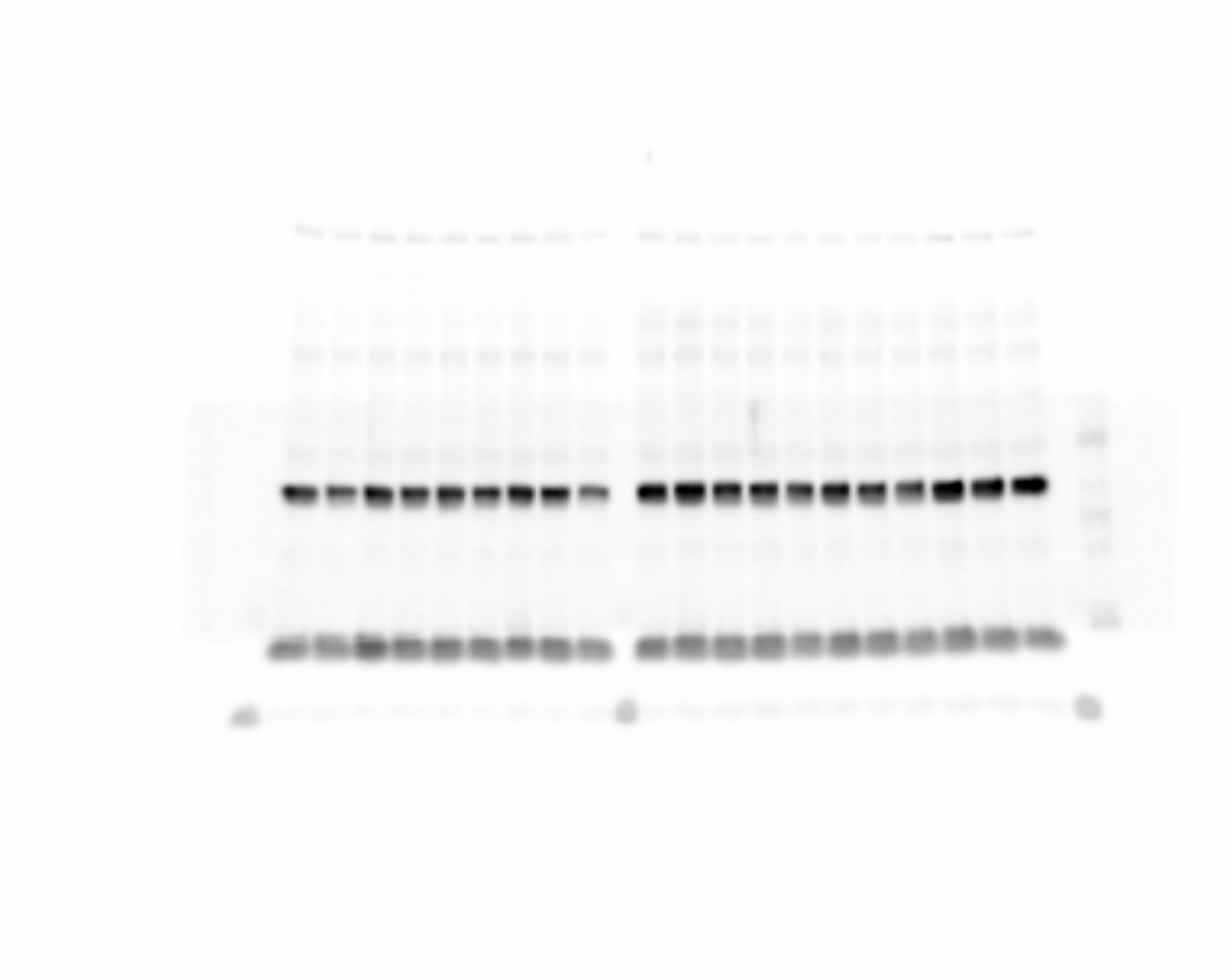


M M M F F F F F F F F

CO CO CO CO CO CO CR CR CR CR CR

~42 kDa

~53 kDa

**≠**


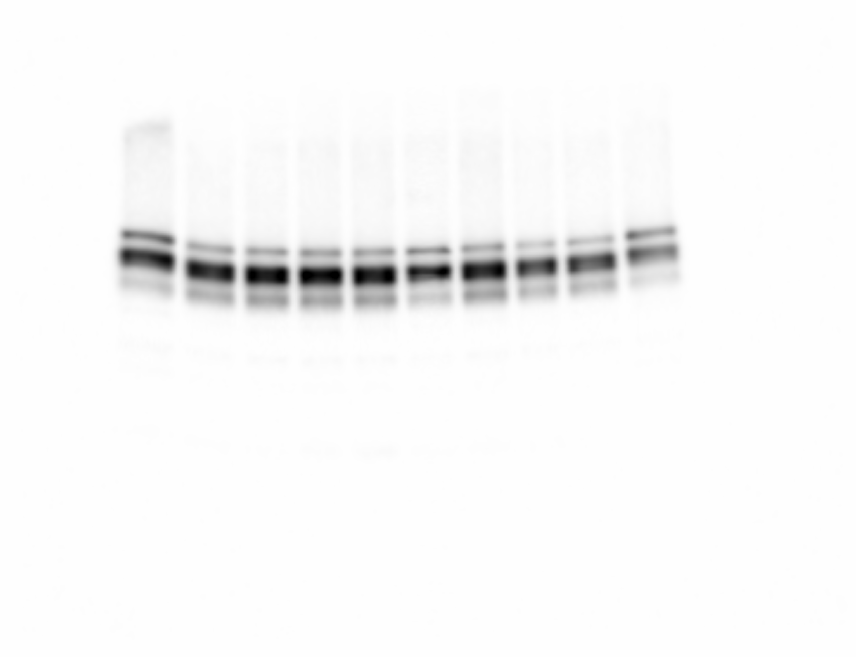

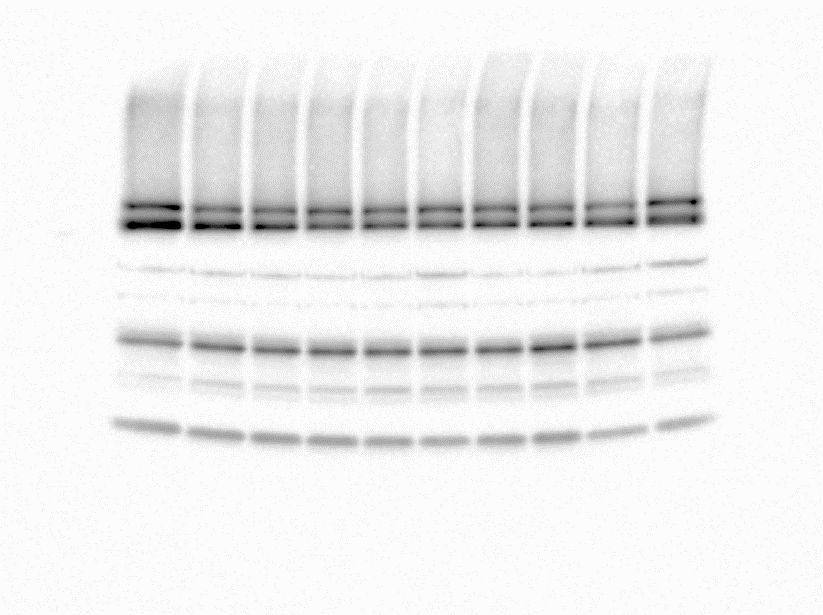
(I) Drp1 (J) Porin

M M M F F F F F F F

CO CO CO CO CO CO CR CR CR CR

M M M F F F F F F F

CO CO CO CO CO CO CR CR CR CR

~82 kDa

~70 kDa

**≠**

**≠**

~33 kDa

(K) Complex I – V

M M M F F F F F F F

CO CO CO CO CO CO CR CR CR CR


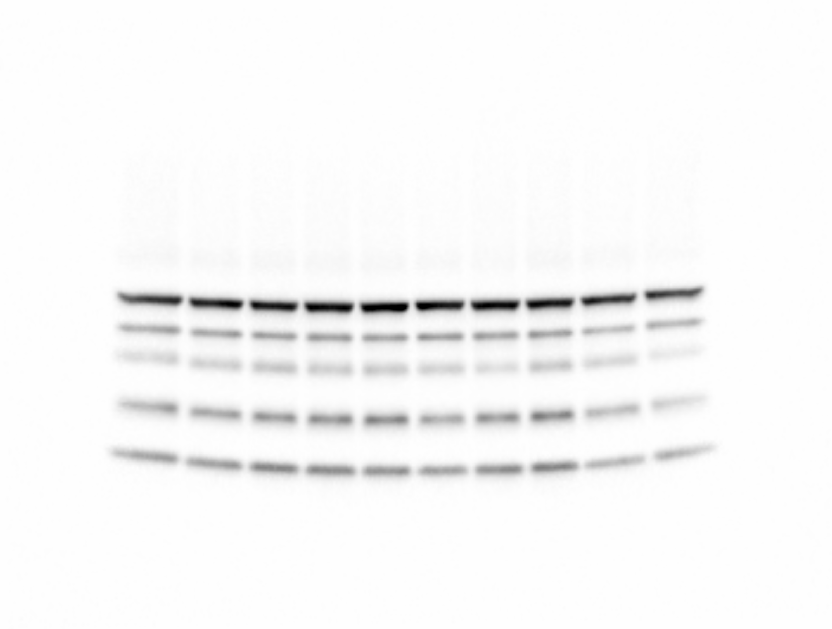


~55 kDa

~40 kDa

~25 kDa

~18 kDa

CV-ATP5A

CIV-MTCO1

CII-SDHB

CI-NDUFB8

~46 kDa

CIII-UQCRC2

**Supplementary Figure 2**. **Western blot membranes for detection of AD-related proteins in 3xTg hippocampus.** Western blot membranes of hippocampal homogenates from 3xTg mice with antibodies (A, B) 4G8 at multiple exposure times to reveal relevant bands, (C) mOC64, (D) A11, (E,F) mOC87 at multiple exposure times, and (G) AT8 for hyperphosphorylated tau. (H) Total tau. M = male; F = female; CO = control; CR = creatine; *C57 sample not part of experiment and not included in statistical analysis.


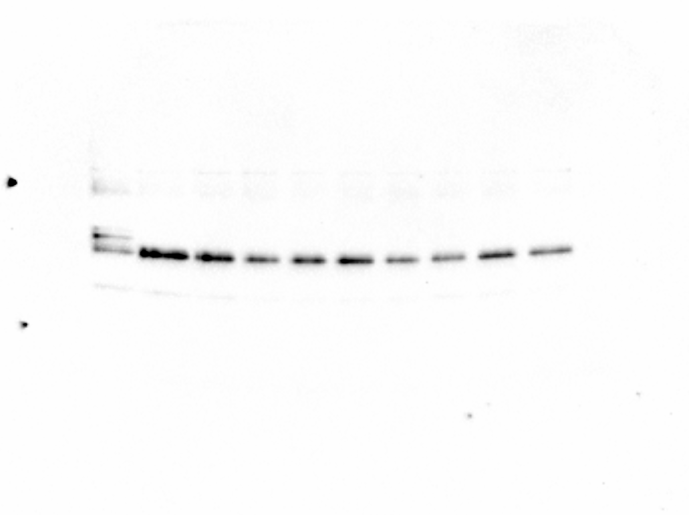

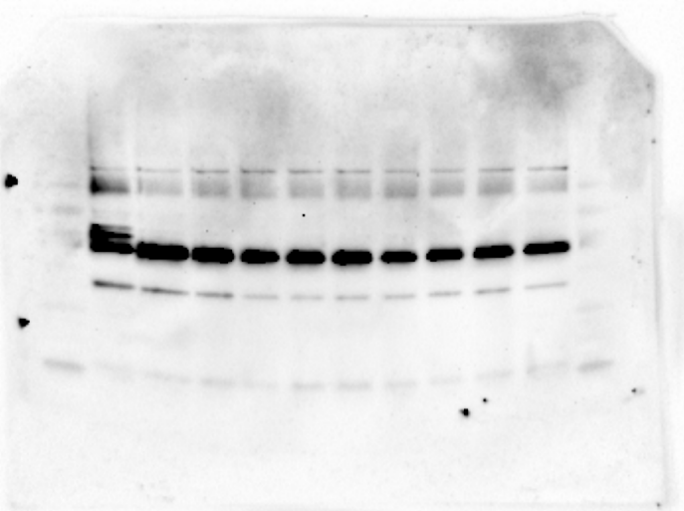
(A) 4G8 – 56 kDa exposure (B) 4G8 – 40 & 100 kDa (APP) band exposure

M M M F F F F F F F

CO CO CO CO CO CO CR CR CR CR

M M M F F F F F F F

CO CO CO CO CO CO CR CR CR CR

40 kDa kDa

100 kDa kDa

56 kDa


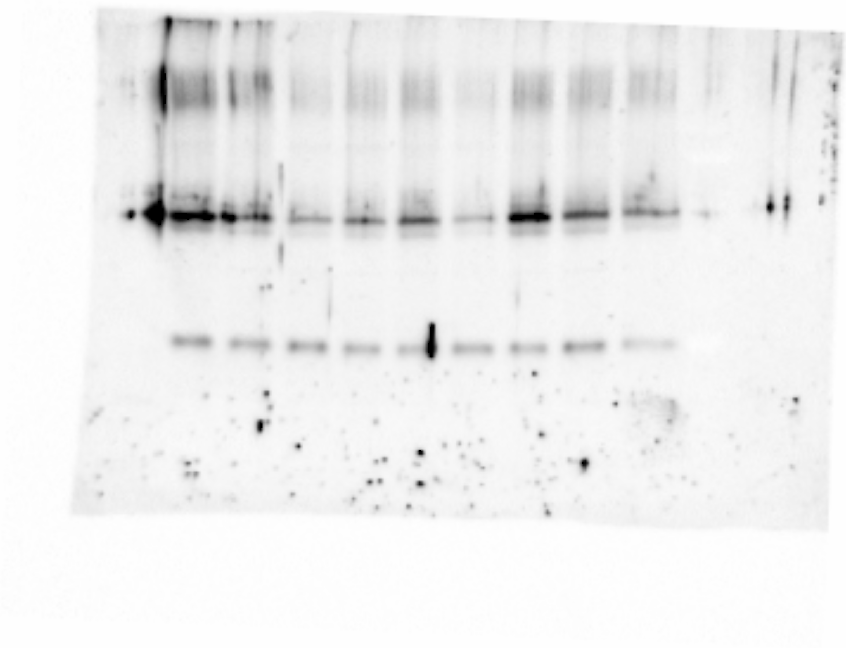

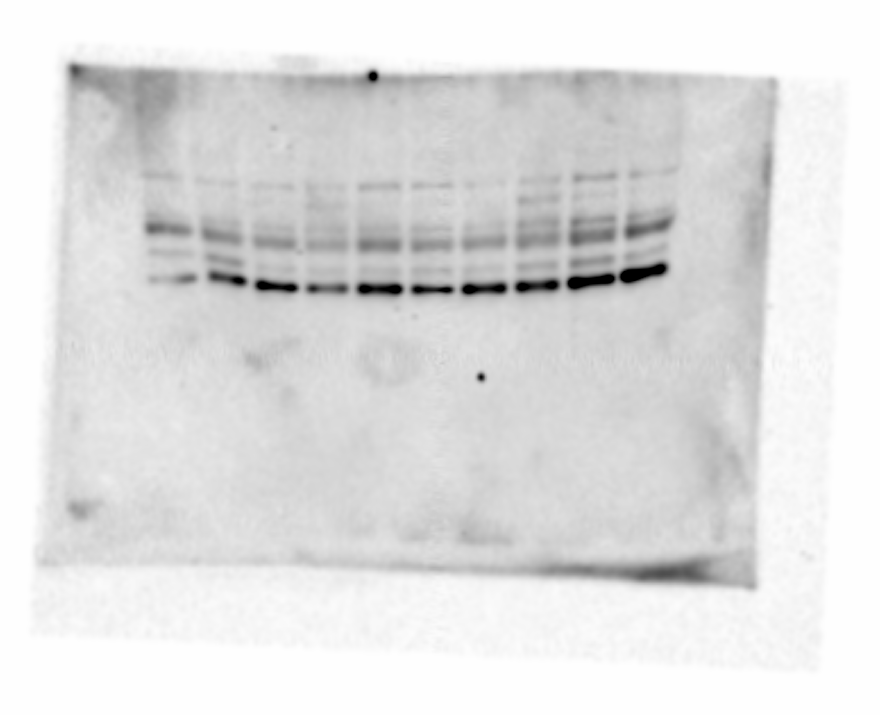
(C) mOC64 (D) A11

72 kDa

64 kDa

56 kDa

48 kDa

40kDa

M M M F F F F F F

CO CO CO CO CO CO CR CR CR

*C57 M M M F F F F F F

CO CO CO CO CO CO CR CR CR

64 kDa

24 kDa

*
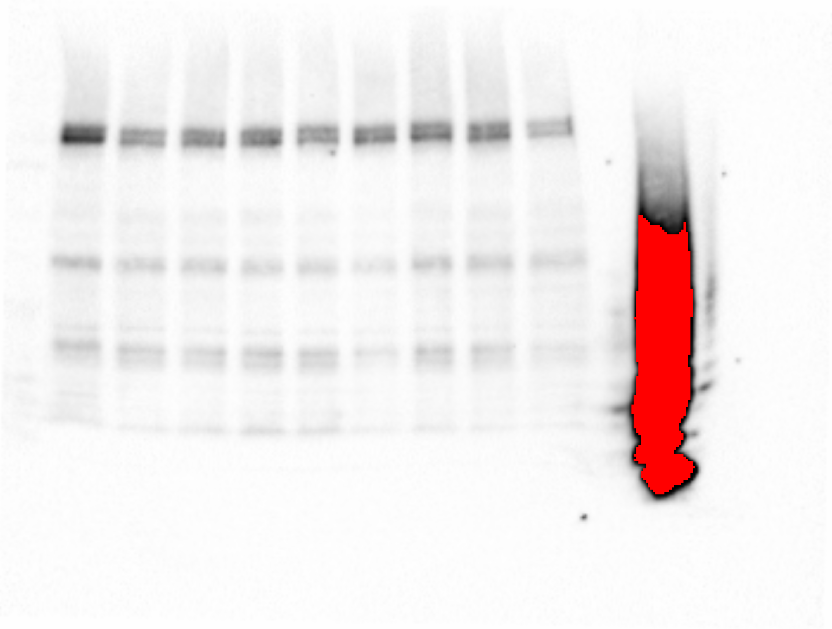

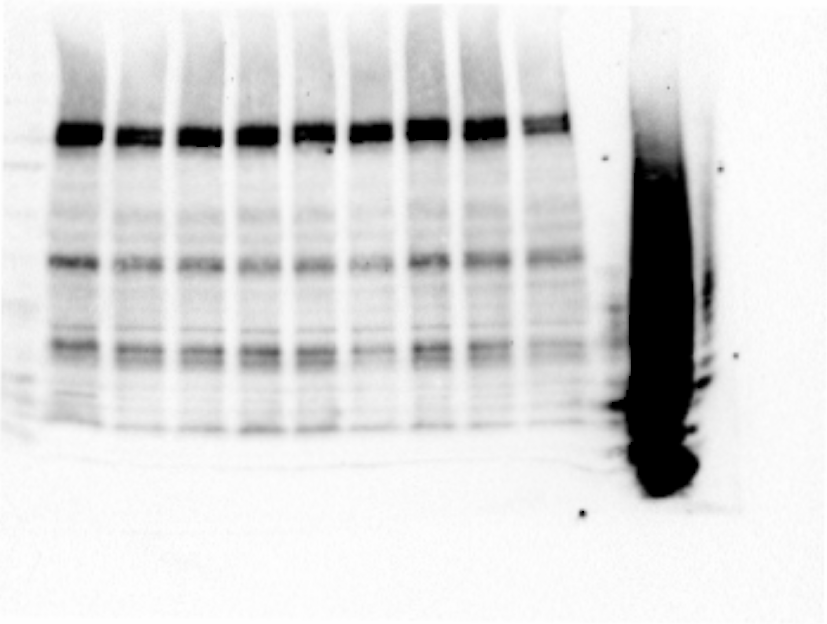
*(E) mOC87 – APP exposure (F) mOC87 – Trimers & HMW Oligomer exposure

M M M F F F F F F

CO CO CO CO CO CO CR CR CR

100 kDa kDa

M M M F F F F F F

CO CO CO CO CO CO CR CR CR

48 kDa

24 kDa kDa

Trimers – 12 kDa kDa

(G) AT8 - ptau (H) Total tau


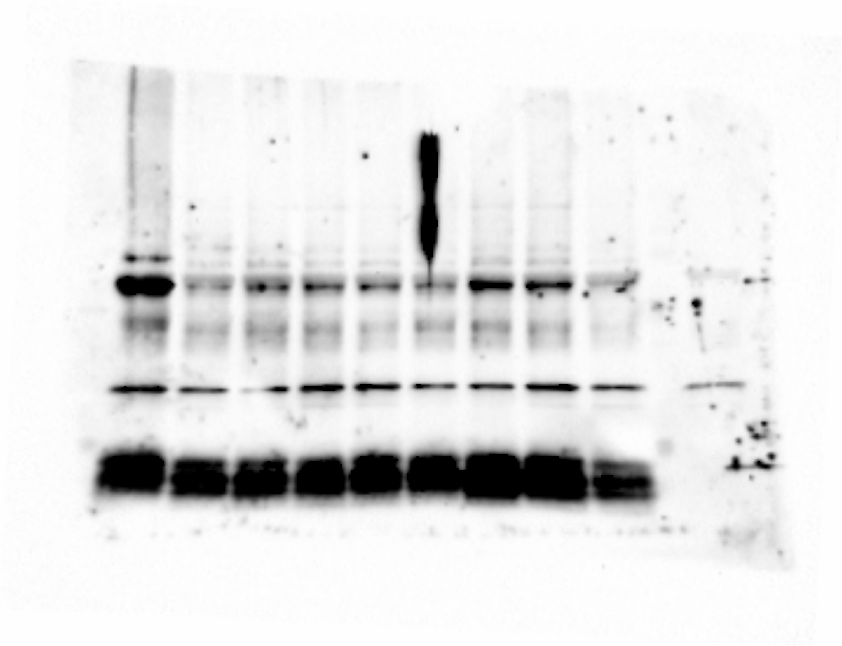

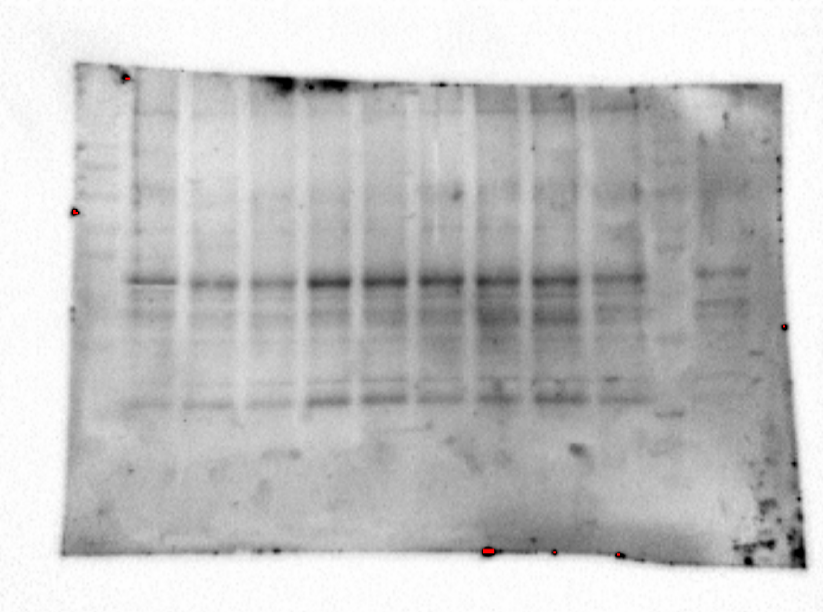


M M M F F F F F F

CO CO CO CO CO CO CR CR CR

M M M F F F F F F

CO CO CO CO CO CO CR CR CR

~55 kDa

~55 kDa
